# Volatile and Sensory Profiles of Young Red Wines Treated with Yeast and Grape Polysaccharides After Malolactic Fermentation

**DOI:** 10.3390/foods15091560

**Published:** 2026-05-01

**Authors:** María Curiel-Fernández, Estela Cano-Mozo, Belén Ayestarán, Zenaida Guadalupe, Thierry Doco, Silvia Pérez-Magariño

**Affiliations:** 1 Grupo de Enología, Instituto Tecnológico Agrario de Castilla y León, Consejería de Agricultura y Ganadería, Ctra. Burgos Km 119, Finca Zamadueñas, 47071 Valladolid, Spain; mariacuriel2a@gmail.com (M.C.-F.); ita-canmozes@itacyl.es (E.C.-M.); 2 Instituto de Ciencias de la Vid y el Vino, Universidad de La Rioja, Gobierno de La Rioja, CSIC, Finca de La Grajera, Ctra. Burgos 6, 26007 Logroño, Spain; belen.ayestaran@unirioja.es (B.A.); zenaida.guadalupe@unirioja.es (Z.G.); 3 Unité Mixte de Recherche Sciences Pour l’Œnologie, Institut Agro, University Montpellier, 2 Place Viala, F-34060 Montpellier, France; thierry.doco@inrae.fr

**Keywords:** volatile compounds, polysaccharides, wine quality, wine composition, aroma perception, sensory analysis, astringency

## Abstract

Volatile compounds contribute to wine aroma and can interact with polyphenols, polysaccharides (PS), and proteins. This work evaluated the effects of adding different PS extracts obtained from winery by-products, must and wine on the volatile composition and sensory attributes of young red wines. These results highlight the effect of the wine matrix on the impact of PS on the volatile composition. The highest concentrations of volatile compounds were reached in wines with higher phenolic content and mainly those treated with PS extracts contained higher proportions of low-molecular-weight PS (55–68%). These PS extracts maintained high concentrations of compounds related to fruity and floral aromas, such as ethyl esters of fatty acids (8–23%), alcohol acetates (9–23%), and terpenes (11–43%). In addition, the PS extracts from winery by-products and wines improved taste sensations in young red wines, mainly those with high acidity, by reducing acidity, bitterness and astringency. Therefore, PS extracts obtained from by-products have the capacity to modulate the volatile composition and mouthfeel of red wines with high phenolic content, excess acidity or astringency.

## 1. Introduction

The perceived quality of wine is closely associated with its visual, olfactory and gustatory attributes. The wine aromatic profile is constituted by chemical compounds with different volatilities and perception thresholds. These compounds are classified into different groups based on their chemical properties, such as alcohols, ethyl esters of fatty acids, ethyl esters of acetic acid, aldehydes, volatile phenols, acids and terpenes. Their combination is responsible for the distinctive aroma of wine [1]. Volatile compounds provide a range of characteristic odors, including floral, fruity, herbal, toasted, animal, among others. Additionally, certain macromolecules within the wine matrix such as proteins, polyphenols and polysaccharides can affect the physico-chemical properties of volatile compounds [2,3,4], as well as other taste characteristics such as bitterness and astringency [5,6,7].

Polysaccharides (PS) exhibit colloidal behavior and play a protective role in wine [8]. According to their composition, grape PS can be categorized into the following families: PS rich in arabinose and galactose (PRAG), which include arabinogalactans (AG), arabinans (Ar) and arabinogalactan proteins (AGP); two types of rhamnogalacturonans (RG-I and RG-II), homogalacturonans (HG) and non-pectic PS (NPP) such as celluloses and hemicelluloses [9]. The PS derived from yeast are mannoproteins (MP) and glucans (GL).

Several studies have demonstrated the impact of PS on the technological and sensory attributes of wines [6,10,11,12,13,14,15,16,17,18]. Most of these studies have used PS derived from yeast rich in MP, due to their oenological importance in lees aging processes and their commercial availability [4,10,11]. These studies have shown that MP can interact with certain aroma compounds, affecting their retention or release, and thereby influencing the aroma of wines [4,10,11]. These authors also demonstrated that these interactions can be reversible, depending on the structure and conformation of MP, or the purity of the commercial products used [4,10,11]. The interaction between PS and volatile compounds is complex, involving hydrogen bonding and hydrophobic mechanisms. The strength of these interactions may determine whether volatile compounds are retained or released [14,15,16]. These changes, together with the interaction of PS with phenolic compounds, also modify the sensory perception of wine. MP-rich extracts incorporated into wine additionally contribute to the stabilization of colloidal compounds and color matter in wines and modulate astringency [12,13]. Furthermore, mannoproteins have been associated with improvements in the mouthfeel of wines, including increased viscosity, smoothness and volume perception [17]. Research consistently demonstrates the influence of the structure and composition of MP on the effects observed in wines, as they determine the accessibility within the PS and other wine compounds, predominantly polyphenols. Therefore, the structure and protein content of MP influence the molecular mechanisms affecting the interaction between flavanols and salivary proteins, consequently modulating astringency [13,18,19].

The interactions between grape PS and the phenolic compounds (mainly anthocyanins, tannins and flavonols) and volatile compounds (mainly ethyl esters of fatty acids and alcohol acetates) present in wine have been less studied, probably due to the challenges associated with extraction and the lack of commercially available products. Jiménez-Martínez et al. [5] investigated the impact of cell wall material (CWM) extracted from red pomaces (*Vitis vinifera* L. *Monastrell* and *Cabernet sauvignon*) with boiling water and mixed with ethanol. They also studied their interactions with anthocyanins and tannins showing a reduction in tannin content, astringency and bitterness, concluding that this material could potentially be used as a fining agent. Alternatively, Manjón et al. [6] studied soluble CWM obtained from white and red grape skins *Vitis vinifera* L. *Muscat* and *Tempranillo* by extraction with boiling water and the addition of ethanol. They observed that red grape CWM can interact with flavonols and may modulate wine astringency, which is related to PS composition. Red grape skins contained higher amounts of AG and high molecular weight macromolecules, but lower amounts of Ar than white grape CWM. Similar findings were reported by Brandão et al. [7], who studied the interactions with procyanidins and revealed the potential of two isolated PS fractions from white grape skins (from *Vitis vinifera* L.) to modulate the astringency perception in wines. These two fractions were obtained: one extracted using hot water and the other using ammonium oxalate. As with MP, it is important to note that the effects of the different grape PS extracts on astringency modulation in wines depends on protein structure, the type of PS and other conditions, such as temperature and pH [13,20]. The effect of grape PS on volatile compounds has been less studied. Dufour and Bayonove [2] and Mitropoulou et al. [21] reported a significant relationship between the concentration of purified extracts isolated from wine of AG, AGP and MP and the fluctuation in the release of some volatile compounds such as ethyl octanoate and decanoate, isoamyl acetate and ethyl hexanoate in a model wine. In a recent study carried out by our group, PS extracts from grape pomace were added to white wines during aging, resulting in an increased concentration of volatile compounds related to fruity and floral aromas [22]. Canalejo et al. [23] also used PS extracts derived from grape pomace but as a fining agent in white wine, showing a positive effect on the volatile profile of the wine after one month of bottling due to the presence of MP, GL and NPP.

In the European Union, oenological additives and practices are regulated under Commission Delegated Regulation (EU) 2022/68 [24], which authorizes the use of mannoproteins and other yeast-derived products such as yeast cell walls, inactive yeast and specific yeast preparations. MP and arabic gum obtained from acacia are currently the polysaccharide-based additives used as stabilizers that are recognized by the International Oenological CODEX of OIV and EU regulation [25].

On the other hand, wineries produce large amounts of waste, mainly grape marc and pomace, which can be a valuable source of bioactive compounds such as polyphenols, PS, fibers and minerals. Polyphenols are the most widely studied bioactive compounds due to their antioxidant properties and associated health benefits, including their cardioprotective role [26]. However, the extraction of PS from grape sources has been less well-researched. Currently, there is no specific legislation concerning polysaccharide-rich extracts from winemaking by-products. However, under the circular economy framework, the Spanish Law 7/2022 [27] on waste and contaminated soil for a circular economy allows the classification of certain materials produced during winemaking, such as grape pomace/marc, to be classified as by-products rather than waste. This is provided that the established safety and subsequent use criteria are met. This approach could represent an initial step towards the future inclusion of such products being recognized as a winemaking practice in the future. Therefore, grape-derived PS could represent a promising alternative to commercially available yeast-derived products, which are more commonly used, as they are obtained from winemaking by-products, making them a more sustainable option.

Therefore, the aim of this work was to extract grape PS from winery by-products and use them to improve wine quality. PS extracted from different sources, including grape pomace and marc, must and purified extracts from wine, were added to three young red wines with different oenological characteristics to evaluate their effect on the volatile composition and sensory properties of wines. The results were then compared with those obtained from the use of a commercial yeast PS. Additionally, this study may be relevant to winemakers and the wine industry, as it provides initial insights into the use of grape-derived PS to modulate wine aroma and sensory characteristics, while also promoting the valorization of winemaking by-products.

## 2. Materials and Methods

### 2.1. Chemical Reagents and Standard Compounds

Extraction of grape PS was performed with food grade reagents: tartaric acid (E-334, Agrovin, Ciudad Real, Spain), hydrochloric acid 37% (E-507, Panreac, Madrid, Spain) and rectified alcohol from molasses 96 (0110F, Alcoholes Montplet, Barcelona, Spain). Chromatographic-grade reagents were supplied by Riedel-de Haën (Honeywell, Deutschland, Germany) and the other reagents were supplied by Panreac (Madrid, Spain). Type I water was obtained using Autwomatic Plus 1 + 2 GR (Wasserlab, Barbatáin, Navarra, Spain). Helium BIP (99.99%), air zero (99.99%), and Premier plus hydrogen (99.99%) were provided by Carburos Metálicos S.A. (Valladolid, Spain). Volatile compound standards were purchased from Fluka (Buchs, Switzerland), Sigma-Aldrich (Steinheim, Germany), Alfa Aesar (Lancashire, UK) and Extrasynthèse (Genay, France).

### 2.2. Polysaccharide Extracts

Two polysaccharide extracts were obtained from different wine by-products, *Verdejo* white grape pomace (WGP) and *Tempranillo* red grape marc (RGM). Both extracts were obtained using a method developed by our group [28] from by-products resulting from the pressing of grapes during white and red winemaking processes, respectively. Briefly, the WGP and RGM were defrosted and homogenized using an Ultra Turrax T25 digital (IKA, Staufenim Breisgau, Germany). Extractions were then carried out in an ultrasound bath (J.P. Selecta, Barcelona, Spain) under acidic conditions (2.5 g/L of tartaric acid at pH 1) for 30 min. Then, the samples were stirred in an orbital shaker at 50 rpm (Proeti, Madrid, Spain) for 18 h and centrifuged (3500× *g* for 20 min at 4 °C). The resulting supernatants were concentrated fivefold in a rotary evaporator (Büchi, Flawil, Switzerland) at 35 °C and 40 bars. The polysaccharides were precipitated by adding four volumes of cold acidified ethanol (0.3 M HCl 37% in 96% ethanol), after which the samples were incubated for 24 h at 4 °C. The resulting pellets were then freeze-dried. Two other extracts were obtained from must (WM) and wine (WPP). The WM extract was obtained from a commercial white grape juice concentrate, which was produced by partially dehydrating grape must to reach a ºBrix of 65° ± 0.5 at 20 °C, and was provided by GAP (Albacete, Spain). Commercial must was diluted 1:1 with type I water and polysaccharides from must were recovered via precipitation with cold acidified ethanol (0.3 M HCl 37% in 96% ethanol) for 20 h at 4 °C [29]. The WPP is a purified PS extract obtained from freeze-dried polysaccharides extracted from a *Carignan noir* wine by two successive steps of anion exchange performed at the INRAE Centre in Montpellier (France). The detailed extraction process is described in Canalejo et al. [29]. The freeze-drying process was carried out using a Telstar LyoBeta (Barcelona, Spain). Primary drying proceeded at −20 °C for 1 h under a pressure of 200 µbar. Secondary drying began at 0 °C for 2 h, then continued at 12 °C for 18 h, and finally at 15 °C for 4 h, all at the same pressure. The resulting extracts were obtained as a dry powder and were stored in a desiccator at room temperature to keep them airtight and free from moisture. In addition, an inactivated strain of *Saccharomyces cerevisiae* yeast (CIY), selected by Institut Coopératif du Vin (ICV, France) and provided by Lallemand (Logroño, Spain), was used. All these PS extracts were previously characterized by Canalejo et al. [29] and Pérez-Magariño et al. [22]: WM extract mainly contained 40% mannans, 27.9% NPP, and 19% PRAG; WGP contained 66% NPP and 12.6% PRAG; RGM contained 34.5% NPP and 27.8% PRAG; WPP had a high concentration of RG-II (77%); and CIY contained 69.4% MP and 30.6% GL. The content of total proteins and total polyphenols was less than 3%, so it was not significant enough to affect the retention of volatile compounds compared to the content of total proteins and polyphenols present in wine [4,9].

### 2.3. Grape Varieties, Winemaking Process and Wine Treatments

*Juan García* and *Tempranillo* grape varieties were obtained from the Finca Zamadueñas vineyards in Valladolid (Spain), which are characterized by a dry continental climate. Every two years, the vineyards were treated with 10 t/ha of organic fertilizer. Plant protection adhered to ecological standards, utilizing sulfur-based products to combat *Oidium* and copper-based products for *Mildew*, with adjuvants enhancing contact. From the onset of vegetative dormancy in July until harvest, controlled deficit irrigation was implemented weekly at 20% of the total evapotranspiration.

Red wines were produced using the conventional red winemaking method. Initially, the grapes were destemmed, crushed and sulphur was added (0.05 g/L). Then, the grape pomace was inoculated with commercial Saccharomyces cerevisiae ex r.f. bayanus yeasts (0.20 g/L of EnartisFerm Perlage, Enartis Sepsa, Alcázar de San Juan, Ciudad Real, Spain). Alcoholic fermentation took place at a controlled temperature of 24−26 °C, with 2 min punching down daily. The maceration and alcoholic fermentation processes lasted between 10 and 12 days. Once complete, the wines were separated from the solids and pressed. After 24 h, the wines were racked, and malolactic fermentation was conducted at a controlled temperature of 18−20 °C. After this, the wines were racked again, sulphited (0.04 g/L), and stored at 14–15 °C until they were ready for testing.

These red wines were elaborated and selected for their high phenolic composition, astringency and/or high acidity by sensory evaluation [30]. Wine 1 presented medium-high concentrations of phenolic compounds (1812 mg/L total phenols and 2140 mg/L total tannins) and medium acidity (5.8 g/L tartaric acid); wine 2 presented low amounts of phenolic compounds (1579 mg/L total phenols and 1715 mg/L total tannins) and high acidity (6.6 g/L tartaric acid); and wine 3 had the highest content in phenolic compounds (2566 mg/L total phenols and 3105 mg/L total tannins) and high acidity (6.8 g/L tartaric acid). Wine 1 and wine 2 were from the *Juan García* grape variety and wine 3 from *Tempranillo*.

Seven trials were carried out on each wine in duplicate: control wine (C: no product added); wines with WM extract added (0.2 g/L); wines with two doses of WGP extract added (0.2 g/L and 0.4 g/L); wines with RGM extract added (0.2 g/L); wines with WPP extract added (0.1 g/L); and wines with CIY added (0.2 g/L). The product dose was selected based on the standard recommended doses for commercial yeast products, 0.20 g/L. Given its low polysaccharide concentration, the WGP extract was also assayed at double the dose [22]. The dose of WPP was reduced by half since it is a purified extract with a high polysaccharide concentration [29]. These red wines were in contact with the products for two months at 15 °C ± 1 °C, with two *bâtonnage* per week for 30 s. At the end of this period, the wines were filtered through 0.8 µm cellulose fiber membrane plates, bottled and stored at a controlled temperature (14 °C ± 1 °C) for six months until their analysis. Three bottles of each treatment were analyzed.

### 2.4. Analytical Methods

Volatile compounds were determined by two analytical methods. Higher alcohols were quantified by direct injection of 1 μL of wine into an Agilent 7890A gas chromatograph with a flame ionization detector (GC-FID), using the chromatographic and quantification conditions established by Pérez-Magariño et al. [31]. The samples were injected in split mode (25:1), and the higher alcohols were separated using an Agilent DB-WAX capillary column (30 m × 0.25 mm i.d. × 0.25 μm film thickness). The chromatographic conditions were as follows: helium was used as the carrier gas at a flow rate of 0.7 mL/min; the column temperature program was as follows: held at 40 °C for 4 min, heated at 1 °C/min to 70 °C, then heated at 30 °C/min to 200 °C (held for 10 min); and the injection temperature was 250 °C. Each compound was identified and quantified using calibration graphs built with pure standard solutions analyzed under the same conditions (Supplementary Table S1). In addition, reference materials were used to ensure the precision, accuracy, and traceability of the results.

Minor volatile compounds were extracted by liquid–liquid extraction with dichloromethane (purity of 99.9%). Two hundred and fifty milliliters of wine, 5 mL of dichloromethane and 75 µL of a mixture of two internal standards (450 mg/L of 3,4-dimethylphenol and 550 mg/L of methyl octanoate) were added in a flask. The extraction was carried out for 2 h with continuous stirring (150 rpm) in an orbital shaker. The organic phase was then separated and concentrated to 400 μL. The analysis was performed by injecting 1 μL of the concentrated extract into a HP-6890N gas chromatograph coupled with a HP-5973 inert mass detector (GC-MS). Chromatographic analyses were performed with an Agilent DB-WAX Ultra Inert capillary column (60 m length, 0.25 mm i.d., and 0.25 mm film thickness), using the chromatographic conditions described in Perez-Magariño et al. [31]. The carrier gas was helium at 0.8 mL/min. The oven column program was set to 40 °C (held for 10 min), raised to 230 °C by 2 °C/min and held at this temperature for 35 min. Detection was in EI mode (70 eV) and identification was carried out using spectra and retention times obtained with commercial standard compounds. Quantification was performed according to the internal standard quantification method, using the quantification ions and internal standards selected for each compound indicated in Supplementary Table S2. Then, quantitative data of the relative areas (absolute areas divided by the internal standard area) were plotted on the calibration graphs built from results for each pure standard compound (Supplementary Table S2). These analyses were carried out in triplicate.

### 2.5. Sensory Analyses of Wines

The sensory analyses were carried out in a test room designed in accordance with the ISO 8589 Standard (2010) and were performed by six expert tasters who were trained previously during 20 training sessions to quantify the defined descriptors, as indicated in Del Barrio-Galán et al. [32]. A structured five-point numerical scale was used, with 1 corresponding to very low intensity and 5 to high intensity for each attribute. Samples were blind-tasted and presented in standard glasses in random order, with each sample coded with a three-digit number. Water was provided to rinse the mouth between evaluations. The questionnaire consisted of 16 sensory descriptors grouped into four visual descriptors (color intensity, purplish tones, red tones and orange shades), five olfactory descriptors (olfactory intensity, red fruit, black fruit, ripe fruit, and yeast) and seven gustatory descriptors (bitterness, acidity, sweetness, astringency, balance, mouthfeel and persistence).

### 2.6. Statistical Analyses

Multivariate analysis of variance (MANOVA) was carried out to show the effects of the wine type, the treatment, and their interaction for each volatile compound evaluated. One-way analysis of variance (ANOVA) and Tukey’s Honestly Significant Difference (HSD) were performed to determine the effects of the treatments on the volatile compounds of the studied wines. Both analyses were carried out at a significance level of *p* < 0.05. The MANOVA and ANOVA analyses were carried out with the Statgraphics Centurion XVIII statistical package (The Plains, Virginia, version 18.1.06). Principal Component Analyses (PCA) was also carried out to study the correlation between the variables and the different wines studied. A hierarchical cluster analysis (HCA) was performed using the Ward agglomerative method and the Euclidean distance metric to evaluate the dissimilarity and grouping of the samples. General Procrustes Analyses (GPA) were performed on the sensory results of the wines to reduce the scaling effects of the different tasters. HCA, PCA and GPA were performed using the XLSTAT software (Addinsoft, version 2023.1).

## 3. Results and Discussion

### 3.1. Volatile Compounds

First, a MANOVA was performed to study the contribution of the wine type and treatment effects in each volatile compound (Table S3). Both factors and their interaction showed a significant influence on most of the compounds, although the wine factor was the primary contributor to the total variance showed by the majority of the compounds studied. Only the interaction between wine type and treatment showed the highest contribution to the variance in four compounds, 1-butanol, *trans*-3-hexen-1-ol, guaiacol, and 4-vinylguaiacol. This indicates that the initial characteristics of wine strongly influence the effects detected in the volatile compounds. Consequently, the effect of treatments was analyzed separately for each wine to consider its initial characteristics.

ANOVA performed on the volatile compounds in the three wines prior to treatment demonstrated statistically significant differences between them. The main volatile compounds in the three wines were the higher alcohols, γ-butyrolactone and fatty acids, followed by the C6 alcohols, ethyl esters of straight-chain fatty acids and alcohol acetates (Table S4). The concentration of most of the volatile compounds was greater in wine 3 than in wine 1 and wine 2, highlighting the ethyl esters of straight-chain fatty acids, ethyl esters of branched-chain fatty acids, alcohol acetates, fatty acids and γ-butyrolactone. Conversely, wine 1 and wine 2 presented the highest values of terpenes and vanillin derivatives. As mentioned above, wine 1 and wine 2 were made using the *Juan García* grape variety, while wine 3 was made with the *Tempranillo* grape variety. Thus, the differences observed could be explained by the grape variety as a key influencing factor [33].

Table 1, Table 2 and Table 3 show the data on the volatile compounds of the three different wines treated with the PS extracts. Wine 1 showed statistically significant differences in most of the volatile compounds analyzed except for the higher alcohols, *cis*-3-hexen-1-ol for C6 alcohols, citronellol for terpenes and ethyl vanillate for vanillin derivatives (Table 1). In general, the concentration of most of the volatile compounds was higher in the treated wine 1 than in the control wine 1. The highest concentrations of volatile compounds were reached mainly with the WPP, WGP1 and CIY treatments.

Wine 2 also showed statistically significant differences in the majority of the volatile compounds between the treatments, with the exception of some higher alcohols, vanillin derivatives and volatile phenols (Table 2). The WGP1 treatment maintained the highest content of ethyl esters of straight-chain fatty acids and alcohol acetates, followed by WM treatment. Additionally, WM showed the highest levels of terpene concentration. Contrary to wine 1, the other treatments had the same or lower content of volatile compounds than the control wine 2. The WPP treatment had the highest total higher alcohol content due to the high concentration of 1-propanol.

Statistically significant differences were found in the volatile compounds for wine 3, except for C6 alcohols, higher alcohols, ethyl butyrate for EE-SFCA, hexyl acetate and 2-phenylethyl acetate for alcohol acetates, γ-nonalactone and guaiacol for volatile phenols (Table 3). The treatments with WM, WGP1, WPP and CIY led to maintaining high concentrations in ethyl esters of straight-chain fatty acids, ethyl esters of branched-chain fatty acids, alcohol acetates, fatty acids, terpenes and vanillin derivatives. However, all treatments showed a decrease in γ-butyrolatone and volatile phenols.

It is well known that PS found in grapes and yeasts plays a key role in the interaction with certain volatile compounds in wines. However, these interactions are complex and depend on both the type and structure of the PS and the physico-chemical structure of the volatile compound [21,34], as well as on the properties of the wine matrix [11,22].

In general, the treatments carried out in this study with different PS extracts did not significantly affect the content of higher alcohols, which is consistent with previous results obtained by our research group in different varietal white wines [22,23]. Only the wine 2 treated with WM showed a high content of 1-butanol, while the wines treated with WGP, WPP and CIY exhibited low concentrations of 2-phenylethanol. Nevertheless, the concentrations of these compounds were below the threshold levels at which they are considered to have an unpleasant odor. Mitropoulou et al. [21] studied the effect of AG on the release of some aromatic compounds, including higher alcohols, into the headspace of a model wine solution. They showed that the addition of AG at low concentrations (up to 1 g/L) increased the release of most aromatic compounds, which was attributed to a salting-out effect. However, at higher levels of AG, no changes in volatility in isobutanol, 2-methyl-1-butanol and 2-phenylethanol were observed. Other studies conducted with different commercial yeast derivatives, either in wines or model wines, have reported slight differences in the higher alcohol concentrations [11,34].

Higher effects were detected in the concentrations of ethyl esters of fatty acids, alcohol acetates and terpenes, compounds related to the fruity and floral aroma of wines. A high content of ethyl esters of straight-chain fatty acids, ethyl esters of branched-chain fatty acids and most of the terpenes were found in the wine 1 and wine 3 treated with WGP1, WPP and CIY after two months in contact with the PS products and after six months of bottle aging. The most significant interactions in wines are hydrophobic interactions between PS and volatile compounds, which bind more hydrophobic compounds, such as terpenes and ethyl esters, to the protein fraction of PS, mainly MP and PRAG, including AGP [2,14,21]. These authors demonstrated that adding different types of PS, such as MP, AG and AGP, initially reduced the concentration of most hydrophobic volatile compounds in model wine solutions. However, after several months of aging, some authors observed an increase in the levels of ethyl esters and alcohol acetates in model wine solutions, as well as in white and red wines supplemented with yeast cell walls, commercial yeast derivatives, or purified mannoproteins [11,14,35] also showed an increase in these volatile compounds in white wines treated with natural and commercial lees. In addition, Del-Barrio-Galán et al. [11] found that the release of hydrophobic volatile compounds depended on the composition of the commercial yeast product used. Comuzzo et al. [34] and Rodríguez-Bencomo et al. [10] suggested that simple sugars released from these yeast products can sequester a significant amount of water, thereby enhancing the release of volatile molecules (salting-out effect). Dufour and Bayonove [2] also demonstrated that the AGP and RG-II polysaccharide fractions tend to salt out isoamyl acetate and ethyl hexanoate in a model wine, and Mitropoulou et al. [21] showed that the volatility of more hydrophobic esters (ethyl octanoate, ethyl decanoate, and ethyl dodecanoate) increased with the addition of AG at low concentrations in a model wine. Therefore, over time, the effects of retention and salting-out may become competitive, and the interactions could potentially reverse after extended aging, suggesting that the concentrations of the PS and volatile compounds are also relevant.

On the other hand, Canalejo et al. [23] reported that adding PS extracts from white and red pomace, white must, and white lees increased the content of total ethyl esters of fatty acids and alcohol acetates after one and twelve months of bottling in *Viura* wines. Similar results were obtained by Pérez-Magariño et al. [22], showing that grape PS extracts (WM, WGP) and commercial inactivated yeasts (CIY) increased the concentrations of ethyl esters of straight-chain fatty acids and of alcohol acetates in *Albillo* wines. These results were consistent with those found in this study with red wines. The WGP extract was mainly composed of PRAG and NPP, while white lees and CIY were predominantly composed of MP. Despite these differences, all extracts contained higher proportions of low-molecular-weight PS [22,23], which could interact with the hydrophobic regions of ethyl esters of fatty acids and alcohol acetates, thereby reducing the hydrolysis of these compounds during aging.

Similar results were generally found for terpenoid compounds, with an increase in concentration observed in different white wines treated with PS from either white grape pomace or commercial yeasts [22,23]. This is consistent with the findings of the present research. Bautista et al. [35] also found the highest levels of monoterpenes in white wines matured with commercial lees. In contrast, Del Barrio-Galán et al. [11] indicated that the concentrations of terpenes were minimally affected by commercial yeast derivatives in red wines. In model wine, Mitropoulou et al. [21] found that the addition of AG increased the volatility of linalool at low concentrations. These results suggest that the concentrations of both PS and volatiles are important factors that can affect retention due to competition for binding sites [34].

Regarding the total C6 alcohols, different trends were found in the three wines. The wine 1 treated with WGP2, RGM, WPP and CIY extracts presented the highest concentrations, whereas the wine 2 treated with WGP, RGM and CIY extracts showed the lowest. These compounds did not show statistically significant differences in wine 3 due to the treatment effect. The odor thresholds for the C6 alcohols evaluated are much higher than the concentrations found in these wines, so they will not affect the herbal and grassy notes they provide to the wine. Pérez-Magariño et al. [22] and Canalejo et al. [23] observed an increase in C6 alcohol levels in white wines treated with PS extracted from must, pomace and lees. However, these levels remained below the odor threshold. Conversely, Mitropoulou et al. [21] observed no effect of 1-hexanol volatility at AG concentrations of up to 1 g/L in a model wine solution. Nevertheless, an increase in AG concentration was found to reduce its volatility, suggesting a molecular interaction between 1-hexanol and AG. This is consistent with the results of Dufour and Bayonove [2], who also reported a reduction in 1-hexanol levels with increased PS addition.

As with the ethyl esters of fatty acids and alcohol acetate compounds, the effect of the PS extracts on the fatty acids depended on the type of wine. Treatments with WGP, WPP and CIY increased the concentration of fatty acids in wine 1 and wine 3, but decreased it in wine 2. An increase in fatty acids was also reported in *Verdejo* and *Viura* wines treated with PS extracts from must and pomace during aging in tanks; however, *Albillo* wines were unaffected [22,23]. Del-Barrio-Galán et al. [11] obtained different results depending on the type of fatty acid and commercial yeast derivative used, with no clear effect observed. Other authors have demonstrated that the use of natural lees or commercial lees has a binding effect on certain fatty acids after a period of aging in white and red wines, as well as in model solutions [11,34,35]. In this study, the concentrations of total fatty acids in all wines were below 10,000 µg/L. This suggests that they may contribute to the pleasant aromas of the wines [36] and that slight changes caused by adding PS extracts will not affect the overall aroma.

Only four volatile phenols were detected in the wines under study: guaiacol, 4-vinylguaiacol, eugenol and syringol. These compounds are associated with spicy, smoky or toasted notes [1]. Other volatile phenols, such as 4-ethylphenol and 4-vinylphenol, which are often associated with unpleasant odors like leather, stable or medicine, were not detected in this study. Of all the compounds detected, 4-vinylguaiacol had the highest concentrations and exceeded the odor threshold in all the wines. The effect of adding PS extracts depended on the wine. The wine 1 treated with grape and yeast PS had a higher content than the control wine. In contrast, the wine 3 treated with PS products had a lower content, while wine 2 showed no statistically significant differences. Previous studies carried out by our group with these grape and yeast PS products revealed similar contents or slight reductions in volatile phenols in treated white wines [22]. However, extracts from white pomace and white lees were found to increase the concentration of volatile phenols in *Viura* wines when used as fining agents [23]. Similarly to what was observed in wine 3, other researchers have reported the binding of eugenol, 4-ethylphenol, 4-vinylphenol or syringol in red wines treated with different commercial yeast derivatives or aged on lees [10,11,34]. Chassagne et al. [37] also observed the sorption of 4-ethylphenol by wine lees, indicating that this effect depends on the extent of yeast autolysis and physicochemical parameters such as ethanol content and pH.

Regarding lactones, no statistically significant differences in γ-nonalactone were found due to the effect of PS extracts. However, the addition of the extracts reduced the γ-butyrolactone content in wine 2 and wine 3, while increasing it in the wine 1 treated with WGP, WPP and CIY. The most abundant vanillin derivative compounds were ethyl vanillate. In general, the addition of PS extracts did not modify its concentration in wine 1 and wine 2, but increased it in the wine 3 treated with WGP, WPP and CIY. Very few studies analyzing the effect of grape and yeast PS on lactones and vanillin derivatives were found. Del Barrio-Galán et al. [11] observed no significant differences in lactone or vanillin derivative content, except for ethyl vanillate, which increased significantly in red wines treated with commercial yeast derivatives or aged on lees. Bautista et al. [35] demonstrated an increase in the content of ethyl vanillate and acetovanillone in white wines that were aged with natural lees or commercial lees for seven months.

It should be noted that, in general, increasing the dose of WGP did not improve the volatile composition. The effects observed in wine 1 and wine 3 treated with WGP, WPP and CIY were not as clear in wine 2. Therefore, these results highlight the effect of the wine matrix on the impact of PS on the volatile composition. The non-volatile matrix, which consists mainly of polyphenols and PS, plays a crucial role in determining aroma perception. This is because it strongly influences the volatility of aromatic compounds, which can be retained or released via the salting-out effect [10,38]. The interactions between these compounds are typically hydrophobic, but also involve hydrogen bonding, van der Waals forces, and π–π stacking between aromatic rings [39,40]. The strength and nature of these interactions depend on the specific structural characteristics of all the compounds. Polyphenols interact more strongly and preferentially with PS than with volatiles, producing significant effects on astringency and mouthfeel sensations, as discussed below. However, they may also influence aroma indirectly. Most of these studies have been conducted in hydroalcoholic solutions, model wines or reconstituted wines. Compounds such as catechins and tannins have been shown to retain certain volatiles, including esters [3] and fatty acids [38], among others, thereby reducing their volatility. Nevertheless, other authors have reported an increase in volatile compound concentrations in the presence of higher levels of phenolic compounds, depending on their molecular structure [10,21]. Therefore, adding PS to wines with high polyphenolic content (such as wine 1 and wine 3) could reduce interactions between phenolic compounds and volatile compounds, enabling the latter to be released [41,42].

### 3.2. Multivariate Statistical Analyses

**Figure 1:**
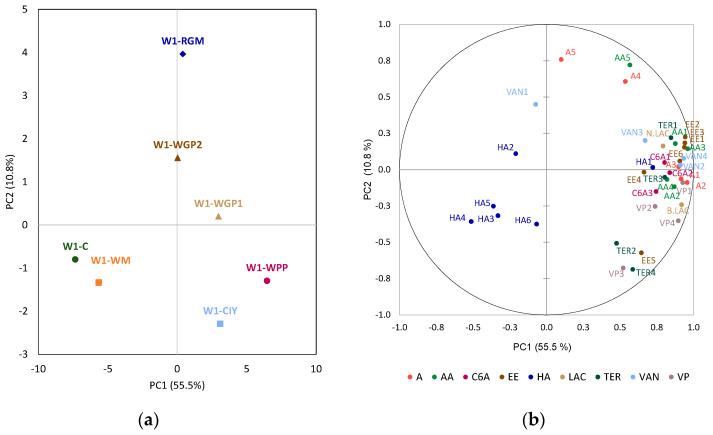
Principal component analysis: (**a**) distribution of the wine 1 (W1) treatments; (**b**) loadings of the volatile compounds analyzed. C: wine control; WM: wine with white concentrated must extract; WGP1 and WGP2: wine with 0.2 g/L and 0.4 g/L of white grape pomace extract, respectively; RGM: wine with red grape marc extract; WPP: wine with a wine-purified PS extract; CIY: wine with an inactivated commercial yeast. Abbreviations of compounds in Table 1.

**Figure 2:**
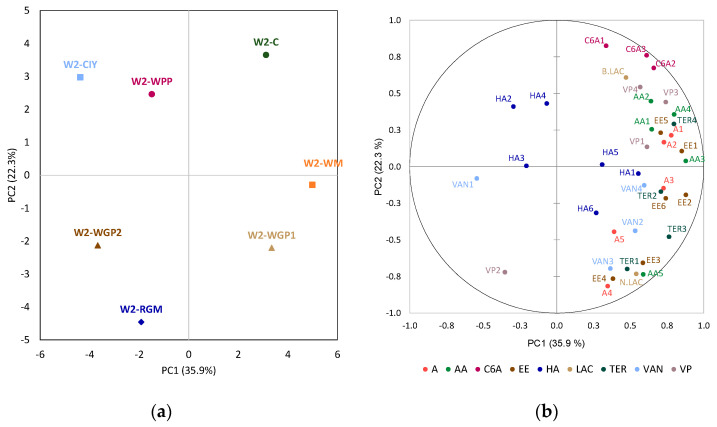
Principal component analysis: (**a**) distribution of the wine 2 (W2) treatments; (**b**) loadings of the volatile compounds analyzed. C: wine control; WM: wine with white concentrated must extract; WGP1 and WGP2: wine with 0.2 g/L and 0.4 g/L of white grape pomace extract, respectively; RGM: wine with red grape marc extract; WPP: wine with a wine-purified PS extract; CIY: wine with an inactivated commercial yeast. Abbreviations of compounds in Table 1.

**Figure 3:**
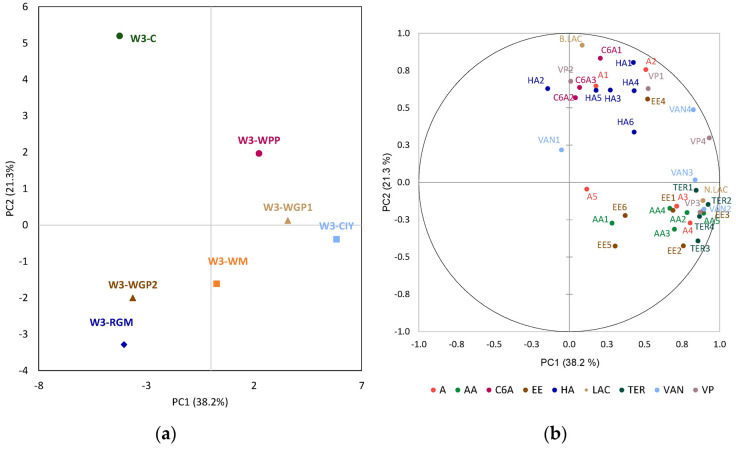
Principal component analysis: (**a**) distribution of the wine 3 (W3) treatments; (**b**) loadings of the volatile compounds analyzed. C: wine control; WM: wine with white concentrated must extract; WGP1 and WGP2: wine with 0.2 g/L and 0.4 g/L of white grape pomace extract, respectively; RGM: wine with red grape marc extract; WPP: wine with a wine-purified PS extract; CIY: wine with an inactivated commercial yeast. Abbreviations of compounds in Table 1.

Principal Component Analysis (PCA) was performed to investigate the correlation between volatile compounds and the treatment applied to each wine (Figure 1, Figure 2 and Figure 3).

For wine 1, the plane defined by the principal components 1 and 2 explained 66.3% of the total variance (Figure 1a). This showed that the wine treated with WM was similar to the C, exhibiting a stronger correlation with higher alcohol content and being characterized by low concentrations of other volatile compounds (Figure 1b). Wine 1 treated with WGP1, WPP and CIY presented the greatest distances from the C and were located in the bottom-right side of the plane. These wines showed a positive correlation with most of the volatile compounds, which were characterized by a high concentration of most of these volatile compounds. Conversely, the wine 1 treated with WGP2 and RGM was located in the upper part of the plane and was characterized by high levels of vanillin, decanoic acid, dodecanoic acid and 2-phenylethyl acetate.

The first two principal components of the PCA for wine 2 explained 58.2% of the total variance and they showed a different distribution from that of the treated wines (Figure 2a). Control wine 2 was distant from the treated wines and showed an association with C6 alcohols and γ-butyrolactone (Figure 2b). The WPP and CIY treatments clustered closely together and showed correlations with higher alcohols. They were characterized by having low concentrations of most of the volatile compounds. In addition, two other groups were identified: one with the wines treated with RGM and WGP2, which were associated with vanillin and 4-vinylguaiacol content, and another with the wines treated with WM and WGP1, and highly correlated with ethyl esters of fatty acids, alcohol acetates and terpenes (Figure 2b).

Finally, Figure 3a shows the distribution of the differently treated wine 3 studied in the plane defined by the first two principal components, which explained 59.5% of the total variance. The control wine 3 was completely separated from the treated wines and there was no close relationship with the volatile compounds studied, indicating that, in general, it had lower concentrations of the volatile compounds than the rest of the treated wines. The wine 3 treated with CIY, WGP1 and WPP presented the greatest distances from the C and were located in the right part of the plane, showing a stronger positive association with many of the families of volatile compounds (Figure 3b). On the other hand, the wine 3 treated with WGP2 and RGM was on the opposite side, showing a negative relationship with most of the volatile compounds.

As it was previously commented in the univariate analyses of each compound, different effects were observed depending on the wine matrix. The levels of many of the volatile compounds, mainly ethyl esters of fatty acids, alcohol acetates and terpenes (compounds associated with fruity and floral aromas in wine), increased in the WGP, WPP and CIY extracts in wine 1 and wine 3. This may be due to the fact that the addition of PS may disrupt the interaction between phenolic and volatile compounds, thereby limiting the retention of volatile compounds within the matrix of wines with higher phenolic content [41,42].

**Figure 4:**
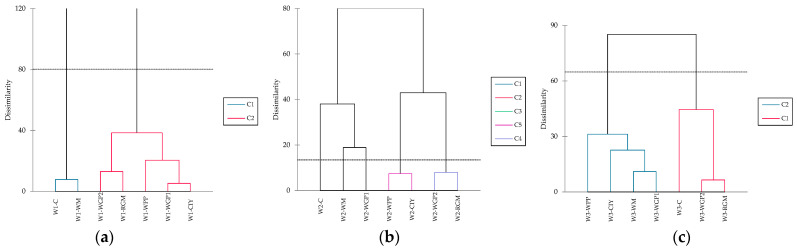
Hierarchical cluster analyses: (**a**) groups of the wine 1 (W1) treatments; (**b**) groups of the wine 2 (W2) treatments; groups of the wine 3 (W3) treatments. (**c**): wine control; WM: wine with white concentrated must extract; WGP1 and WGP2: wine with 0.2 g/L and 0.4 g/L of white grape pomace extract, respectively; RGM: wine with red grape marc extract; WPP: wine with a wine-purified PS extract; CIY: wine with an inactivated commercial yeast.

Hierarchical cluster analyses (HCA) corroborated the patterns revealed by the PCA, confirming the distinction between control and treated wines, as well as the grouping of samples according to the type of PS extract (Figure 4). Two clusters were obtained for wines 1 and 3, while five clusters were formed for wine 2.

### 3.3. Sensory Analyses

There were no statistically significant differences in the visual attributes of the wines by treatment, suggesting that the treatments had no impact on their color. A Generalized Procrustes Analysis (GPA) was carried out with the olfactory and gustative attributes of each wine.

**Figure 5:**
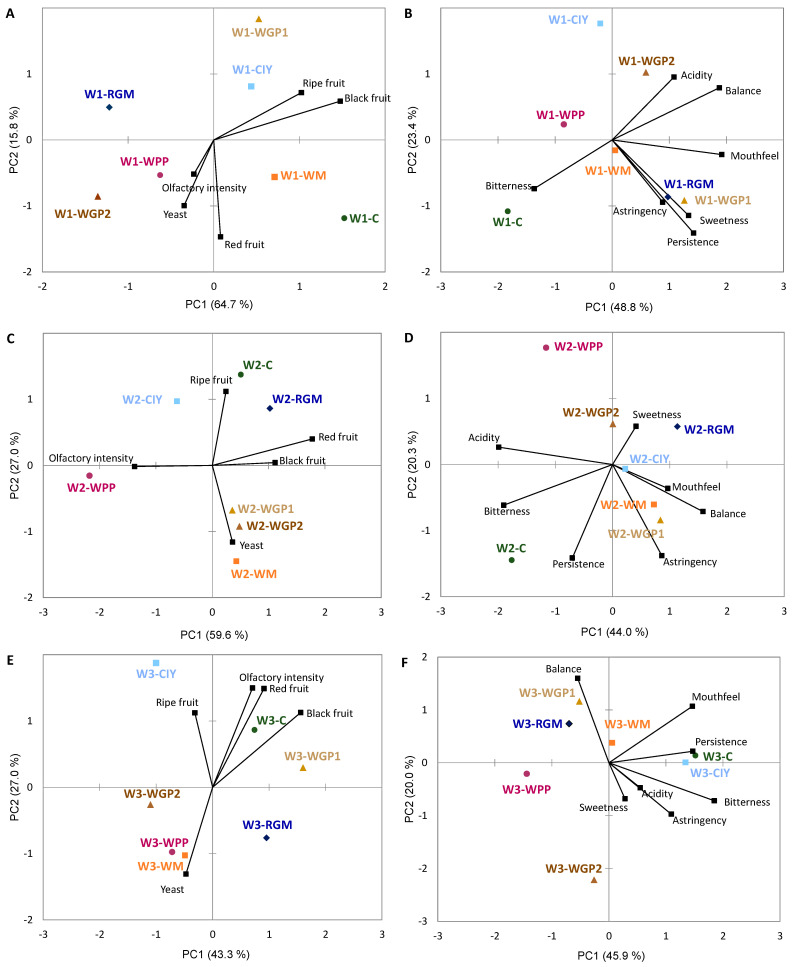
Generalized Procrustes Analysis of the mean ratings for (**A**,**C**,**E**) olfactory phase and (**B**,**D**,**F**) gustative phase in all the wine treatments. C: wine control; WM: wine with white concentrated must extract; WGP1 and WGP2: wine with 0.2 g/L and 0.4 g/L of white grape pomace extract, respectively; RGM: wine with red grape marc extract; WPP: wine with a wine-purified PS extract; CIY: wine with an inactivated commercial yeast.

The GPA consensus configuration for the treated wine 1 explained 80.5% and 72.2% of the total variance in the olfactory and gustative phases, respectively (Figure 5A,B), revealing differences between the treatments. In the olfactory GPA space, the C wine was positioned near the WM-treated wine; both were associated with fruit aromas, particularly red fruit. Conversely, the WGP2 wine was close to the WPP treatment, characterized by olfactory intensity and yeast aroma. The CIY and WGP1 wines were located in the top right quadrant and were related to ripe and black fruit notes. The RGM wine did not stand out for any olfactory attribute. By contrast, the C wine was separated from the other treatments in the gustative GPA space, being associated with bitterness and having lower acidity, mouthfeel and balance than the treated wines. The CIY and WGP2 wines were characterized by acidity and balance, while the RGM and WGP1 wines were characterized by sweetness, mouthfeel, persistence and astringency.

Regarding wine 2, the olfactory GPA space defined by the first two factors accounted for 86.6% of the total variance (Figure 5C). The treated wines were clearly different from the C wine, except for the RGM and CIY wines. These wines were characterized by ripe and red fruit attributes. In the bottom-right corner of the space, the WGP1, WGP2 and WM wines were clustered together and associated with yeast notes and also with red and black fruit. Meanwhile, the WPP wine was placed on the negative side of the space and related to a higher olfactory intensity similar to the trend observed in wine 1. The consensus gustative space explained 64.3% of the variance (Figure 5D). The C wine was clearly separated from the other treatments and it was primarily associated with acidity, bitterness and persistence. The WGP1 and WM wines showed similar characteristics and were characterized by astringency, mouthfeel and balance, whereas the RGM, WGP2 and CIY wines were more closely associated with sweetness.

The GPA consensus configuration for wine 3 explained 70.3% of the total variance in the olfactory space and 65.9% in the gustative space (Figure 5E,F). The C, WGP1 and CIY wines showed a strong correlation with olfactory intensity. The C and WGP1 were related to red and black fruit notes, while the CIY was related to ripe fruit. In contrast, the WM, WPP and WGP2 wines were associated with the yeast aromas. In the gustative space, the C and CIY wines presented high persistence, bitterness and mouthfeel. The WGP1 and RGM wines were more balanced, with lower astringency and acidity. Finally, the WGP2 and WPP presented low mouthfeel, persistence and balance.

Most studies investigating the impact of PS products on sensory aroma attributes have focused on white wines rather than red wines. Rodríguez-Bencomo et al. [10] and Del Barrio-Galán et al. [11] found that different CIY products had no significant effect on the aroma attributes of red wines. However, Rinaldi et al. [43] demonstrated that certain red wines treated with commercial mannoproteins showed increased fruity and floral aromas. Previous studies by our group on white wines have also associated WPP and CIY wines with olfactory yeast and lees characteristics in *Albillo* and *Verdejo* wines [22], while WPP wine from *Viura* was characterized by herbaceous notes [44].

Studies carried out using products rich in PS in red wines have generally focused on gustative attributes such as astringency, bitterness and mouthfeel. In this study, the PS extracted from grape pomace and marc (WGP and RGM) improved taste sensations by reducing acidity, bitterness and astringency, while increasing mouthfeel and balance in the wines with high acidity (wine 2 and wine 3). These PS products also enhanced the mouthfeel and balance of wine 1, reducing bitterness and increasing sweetness. The WPP extract reduced the acidity, bitterness and astringency of the most acidic wines, but had no impact on mouthfeel or balance. These results are consistent with previous findings regarding the use of these PS extracts in white wines [22,44], suggesting that they have the potential to modulate mouthfeel sensations, thereby improving body, sweetness, balance and smoothness. On the other hand, the CIY was less effective in reducing the acidity, bitterness and astringency of the studied wines than the grape PS extracts. Different results have been reported in the literature. Gawel et al. [45] found that adding different PS had no effect on the bitterness, acidity and sweetness of white wines. However, most authors generally observed a reduction in bitterness and astringency, as well as an increase in sweetness and mouthfeel attributes when using MP, PRAG or commercial yeast derivatives [11,22,43,44]. The reduction in bitterness may be attributed to the formation of hydrogen-bonded complexes between PS and bitter phenols [46], which can sterically hinder the interaction of these phenols with taste receptors.

Astringency is another important sensory attribute of red wine, as its excessive perception can be unpleasant. Astringency is a tactile sensation in the mouth often attributed to the precipitation of salivary proteins by tannins [7,47], which reduces lubrication and increases friction. Different studies have shown that PS has a significant effect on the reduction in astringency in wines. Quijada-Morín et al. [47] highlighted that all PS families (PRAG, RG-II and MP) positively influenced astringency perception in red Tempranillo wines, with RG-II and MP having the strongest influence. They suggested that the reduced astringency perception could be related to the branched structures of these complex polysaccharides and the presence of unusual glycosidic linkages. This agrees with our results, which showed a positive effect of WPP (extract rich in RG-II) on astringency, as well as with Canalejo et al. [44], who also found a decrease in astringency in white wines treated with two RG-II-rich extracts.

Various studies have reported that using commercial MP or yeast derivatives that are rich in MP can modulate astringency [11,13] due to their interaction with tannins. However, the composition and structure of these products, as well as the characteristics of the wine, seem to be important factors in determining the extent to which astringency is reduced in wines [13].

Furthermore, Brandão et al. [7] demonstrated the potential of soluble PS fractions from grape skins to compete with salivary proteins through tannin binding. This inhibits the interactions between salivary proteins and tannins and reduces astringency. Similar results were found by Jiménez-Martínez et al. [5] and Manjón et al. [6], who used different cell wall material (CWM) from white and red grape skins in red wines, which interact with phenolic compounds to modulate astringency. These results align with our findings, since the grape PS extracts (WGP and RGM) reduced the negative astringency sensations in the red wines studied.

## 4. Conclusions

This study highlights the importance of the wine matrix, the molecular structure of volatile compounds, and the type of PS in determining the aroma and sensory perception of wine. The addition of PS extracted from winery by-products to wines with high polyphenolic content could reduce interactions between phenolic and volatile compounds, promoting the release of key aroma compounds such as ethyl esters, alcohol acetates and terpenes associated with fruity and floral notes.

From a sensory perspective, PS extracted from grape pomace and marc by-products can modulate the mouthfeel of red wines with excess acidity, bitterness or astringency. In contrast, the influence of a purified polysaccharide extract rich in RG-II was more limited, affecting only specific taste attributes: decreasing the acidity, bitterness and astringency of high-acid wines.

These findings confirm that using PS extracts obtained from wine by-products is an innovative and sustainable way to improve wine quality and promote the circular economy within the wine industry. In practice, these extracts could be used as an alternative oenological tool for modulating the aromatic profile and mouthfeel during winemaking or aging. However, further studies are needed to better understand the interactions involved.

## Figures and Tables

**Table 1:** Volatile compounds analyzed in wine 1 and its treatments ^a^.

Compounds	Abbreviations	Wine 1_C ^b^	Wine 1_WM	Wine 1_ WGP1	Wine 1_ WGP2	Wine 1_ RGM	Wine 1_WPP	Wine 1_CIY	Unit	*p*-Value
2-phenylethanol	HA1	15.3 ± 0.01	15.6 ± 1.14	16.5 ± 0.97	17.5 ± 2.02	16.8 ± 1.1	19.0 ± 0.20	17.0 ± 0.60	mg/L	0.117
1-propanol	HA2	38.9 ± 2.26	38.7 ± 3.50	38.2 ± 2.96	38.9 ± 3.53	38.8 ± 1.33	36.3 ± 0.08	39.4 ± 3.41	mg/L	0.931
Isobutanol	HA3	70.3 ± 1.52	72.6 ± 1.40	67.8 ± 2.73	69.7 ± 1.82	69.4 ± 1.34	71.7 ± 1.37	68.7 ± 3.19	mg/L	0.348
1-butanol	HA4	2.16 ± 0.07	2.27 ± 0.40	1.87 ± 0.04	1.68 ± 0.47	1.71 ± 0.09	1.74 ± 0.33	1.63 ± 0.44	mg/L	0.117
2-methyl-1-butanol	HA5	31.1 ± 1.07	32.8 ± 0.56	30.2 ± 0.38	31.0 ± 0.33	30.3 ± 0.39	31.7 ± 1.34	29.8 ± 1.47	mg/L	0.121
3-methyl-1-butanol	HA6	128 ± 1.31	132 ± 2.05	127 ± 3.23	129 ± 2.92	128.4 ± 1.74	132 ± 0.48	129 ± 2.66	mg/L	0.270
Higher alcohols	HA	286 ± 0.92	294 ± 1.34	282 ± 4.39	288 ± 1.61	285 ± 5.10	293 ± 2.74	286 ± 11.8	mg/L	0.335
Ethyl butyrate	EE1	143 ± 2.48 a	151 ± 3.40 a	170 ± 2.69 bc	168 ± 0.17 bc	166 ± 8.55 b	177 ± 3.18 c	168 ± 2.95 bc	µg/L	**0.001**
Ethyl hexanoate	EE2	169 ± 0.13 a	173 ± 2.83 a	199 ± 3.02 b	196 ± 2.24 b	195 ± 1.49 b	207 ± 4.19 c	194 ± 1.87 b	µg/L	**0.000**
Ethyl octanoate	EE3	128 ± 1.25 a	129 ± 0.12 a	148 ± 0.92 bc	142 ± 0.16 b	150 ± 1.69 c	158 ± 5.95 d	148 ± 2.52 bc	µg/L	**0.000**
Ethyl decanoate	EE4	27.0 ± 0.61 ab	26.6 ± 1.00 ab	28.9 ± 0.68 cd	25.0 ± 0.60 a	30.7 ± 0.12 d	34.1 ± 1.55 e	28.2 ± 1.09 bc	µg/L	**0.000**
Ethyl 2-methylbutyrate	EE5	2.45 ± 0.09 a	2.74 ± 0.00 b	2.94 ± 0.06 c	2.59 ± 0.02 ab	2.44 ± 0.10 a	2.96 ± 0.03 c	2.90 ± 0.05 c	µg/L	**0.000**
Ethyl isovalerate	EE6	5.62 ± 0.25 a	5.93 ± 0.11 ab	6.31 ± 0.37 bc	6.24 ± 0.23 abc	6.52 ± 0.35 bc	6.89 ± 0.29 c	6.68 ± 0.26 c	µg/L	**0.028**
EE-SCFA ^c^	EE-SCFA	467 ± 3.25 a	479 ± 5.36 a	547 ± 5.95 c	531 ± 1.32 b	542 ± 8.22 bc	575 ± 8.51 d	538 ± 8.42 bc	µg/L	**0.000**
EE-BCFA ^c^ Branched esters	EE-BCFA	8.07 ± 0.15 a	8.67 ± 0.11 ab	9.25 ± 0.31 bcd	8.83 ± 0.21 b	8.96 ± 0.45 bc	9.84 ± 0.25 d	9.57 ± 0.31 cd	µg/L	**0.005**
Isobutyl acetate	AA1	64.3 ± 0.49 a	69.6 ± 2.94 ab	78.4 ± 1.56 c	73.63 ± 0.57 bc	74.5 ± 3.86 bc	77.3 ± 1.22 c	75.6 ± 6.13 bc	µg/L	**0.025**
Butyl acetate	AA2	2.54 ± 0.06 a	2.52 ± 0.09 a	2.85 ± 0.05 bc	2.87 ± 0.17 bc	2.66 ± 0.02 ab	2.93 ± 0.09 c	2.89 ± 0.13 bc	µg/L	**0.015**
Isoamyl acetate	AA3	334 ± 6.11 a	345 ± 3.30 a	395 ± 8.98 b	391 ± 4.14 b	384 ± 11.8 b	411 ± 3.76 c	391 ± 1.87 b	µg/L	**0.000**
Hexyl acetate	AA4	1.95 ± 0.13 a	2.17 ± 0.11 b	2.46 ± 0.07 c	2.27 ± 0.03 bc	2.20 ± 0.06 b	2.44 ± 0.05 c	2.32 ± 0.12 bc	µg/L	**0.008**
2-phenylethyl acetate	AA5	10.5 ± 1.02 a	12.6 ± 0.59 ab	15.0 ± 0.34 cd	16.2 ± 0.26 d	18.6 ± 0.21 e	15.4 ± 2.17 cd	13.2 ± 0.36 bc	µg/L	**0.001**
Alcohol acetates	AA	413 ± 7.68 a	431 ± 0.25 a	493 ± 10.9 bc	486 ± 3.17 b	482 ± 15.4 b	509 ± 7.28 c	485 ± 3.90 b	µg/L	**0.000**
Linalool	TER1	6.54 ± 0.32 a	6.76 ± 0.24 ab	7.37 ± 0.09 c	7.32 ± 0.05 c	7.49 ± 0.04 c	7.66 ± 0.08 c	7.27 ± 0.37 bc	µg/L	**0.011**
α-terpineol	TER2	2.11 ± 0.14 ab	2.40 ± 0.05 bc	2.23 ± 0.13 abc	2.05 ± 0.11 a	2.24 ± 0.06 abc	2.53 ± 0.23 c	2.52 ± 0.11 c	µg/L	**0.042**
Citronellol TER	TER3	7.63 ± 0.49	7.81 ± 0.73	8.42 ± 0.37	8.20 ± 0.45	8.41 ± 0.00	8.86 ± 0.53	8.43 ± 0.35	µg/L	0.282
Geraniol	TER4	26.4 ± 0.83 a	26.6 ± 1.63 a	27.0 ± 0.28 ab	25.4 ± 0.45 a	25.5 ± 0.70 a	30.0 ± 2.19 c	29.3 ± 0.47 bc	µg/L	**0.027**
Terpenes	TER	42.7 ± 1.79	43.6 ± 2.55	45.1 ± 0.68	42.9 ± 1.06	43.7 ± 0.59	49.0 ± 3.03	47.6 ± 1.30	µg/L	0.054
1-hexanol	C6A1	1295 ± 91.9 ab	1195 ± 35.8 a	1373 ± 58.9 bc	1435 ± 49.8 bcd	1443 ± 23.4 cd	1557 ± 36.6 d	1489 ± 86.2 cd	µg/L	**0.007**
*trans*-3-hexen-1-ol	C6A2	44.6 ± 0.18 a	44.5 ± 2.23 a	51.8 ± 1.08 bc	50.4 ± 1.12 bc	47.1 ± 2.30 ab	53.0 ± 4.13 c	50.1 ± 0.09 bc	µg/L	**0.021**
*cis*-3-hexen-1-ol	C6A3	30.0 ± 0.23	29.8 ± 1.73	33.4 ± 0.24	31.5 ± 1.33	30.4 ± 0.41	34.0 ± 2.88	32.2 ± 0.55	µg/L	0.098
C6 Alcohols	C6A	1370 ± 91.9 ab	1269 ± 31.8 a	1458 ± 57.6 bc	1517 ± 52.3 cd	1521 ± 20.7 cd	1644 ± 29.5 d	1572 ± 86.9 cd	µg/L	**0.005**
Isovaleric acid	A1	227 ± 0.45 a	228 ± 1.84 a	258 ± 1.87 bc	251 ± 7.91 bc	242 ± 7.33 ab	266 ± 14.3 c	256 ± 5.51 bc	µg/L	**0.005**
Hexanoic acid	A2	899 ± 3.56 ab	892 ± 1.27 a	1021 ± 29.4 de	978 ± 42.0 cd	955 ± 33.3 bc	1057 ± 19.6 e	1013 ± 26.2 cde	µg/L	**0.003**
Octanoic acid	A3	714 ± 10.6	704 ± 24.4	831 ± 60.1	805 ± 37.8	771 ± 46.0	840 ± 14.7	819 ± 59.0	µg/L	0.054
Decanoic acid	A4	210 ± 10.8 a	210 ± 20.1 a	236 ± 11.2 b	237 ± 3.81 b	248 ± 7.26 b	242 ± 8.25 b	207 ± 5.54 a	µg/L	**0.023**
Dodecanoic acid	A5	7.55 ± 0.49 ab	9.30 ± 1.22 cd	8.81 ± 0.55 bcd	8.17 ± 0.31 bc	12.8 ± 0.10 e	9.50 ± 0.08 d	6.74 ± 0.02 a	µg/L	**0.000**
Fatty acids	A	2058 ± 24.0ab	2042 ± 42.7 a	2355 ± 103 cd	2278.51 ± 91.2 cd	2229 ± 79.3 bc	2415 ± 10.9 d	2302 ± 96.2 cd	µg/L	**0.008**
Guaiacol	VP1	3.76 ± 0.00 a	3.75 ± 0.10 a	4.11 ± 0.01 d	3.90 ± 0.11 ab	3.94 ± 0.03 bc	4.19 ± 0.09 d	4.09 ± 0.05 cd	µg/L	**0.002**
4-vinylguaiacol	VP2	51.6 ± 0.18 a	61.2 ± 2.91 bc	67.1 ± 1.29 cd	61.6 ± 3.23 bc	57.6 ± 1.55 ab	70.5 ± 1.72 d	60.6 ± 6.22 bc	µg/L	**0.007**
Eugenol	VP3	11.7 ± 0.44	11.7 ± 1.17	11.8 ± 0.18	11.1 ± 0.10	11.4 ± 0.06	13.04 ± 0.95	13.14 ± 0.22	µg/L	0.063
Syringol	VP4	7.81 ± 0.04 a	7.91 ± 0.13 a	9.22 ± 0.34 b	8.38 ± 0.47 a	8.14 ± 0.14 a	9.54 ± 0.16 b	9.52 ± 0.47 b	µg/L	**0.002**
Volatile phenols	VP	74.8 ± 0.58 a	84.6 ± 4.10 bc	92.3 ± 0.77 cd	85.0 ± 3.99 bc	81.1 ± 1.78 ab	97.3 ± 2.74 d	87.3 ± 5.92 bc	µg/L	**0.005**
γ-butyrolactone	B.LACT	10,279 ± 51.7 a	10,205 ± 453 a	11,499 ± 383 bc	10,650 ± 371 a	10,917 ± 221 ab	12,031 ± 187 c	11,811 ± 359 c	µg/L	**0.003**
γ-nonalactone	N.LACT	22.45 ± 0.63	23.0 ± 2.20	24.8 ± 0.34	24.7 ± 0.18	26.0 ± 0.25	26.3 ± 2.26	25.3 ± 0.18	µg/L	0.115
Lactones	LAC	10,301 ± 52.3 a	10,227 ± 455 a	11,524 ± 383 bc	10,675 ± 371 a	10,943 ± 221 ab	12,057 ± 184 c	11,836 ± 359 c	µg/L	**0.003**
Vanillin	VAN1	16.2 ± 1.34 bc	16.9 ± 2.57 cd	15.3 ± 1.03 abc	13.5 ± 1.32 ab	19.7 ± 0.85 d	18.2 ± 0.74 cd	13.0 ± 0.60 a	µg/L	**0.014**
Methyl vanillate	VAN2	3.96 ± 0.16 a	4.03 ± 0.08 c	4.77 ± 0.25 c	4.54 ± 0.33 c	4.61 ± 0.05 bc	4.83 ± 0.05 ab	4.99 ± 0.38 c	µg/L	**0.014**
Ethyl vanillate	VAN3	354 ± 16.3	398 ± 5.60	397 ± 15.9	410 ± 6.9	410 ± 19.0	411 ± 29.6	410 ± 0.97	µg/L	0.080
Acetovanillone	VAN4	24.0 ± 0.71 a	24.4 ± 0.19 a	27.2 ± 0.46 a	25.5 ± 1.10 b	27.5 ± 0.16 bc	28.8 ± 0.05 bc	27.8 ± 0.97 c	µg/L	**0.001**
Vanillin derivatives	VAN	399 ± 14.1	443 ± 8.28	445 ± 15.6	453 ± 6.79	462 ± 20.1	463 ± 28.9	456 ± 2.91	µg/L	0.053

Mean values ± standard deviation (*n* = 6). Values with different letters in each compound indicate statistically significant differences at *p* < 0.05 (marked in bold). C: wine control; WM: wine with white concentrated must extract; WGP1 and WGP2: wine with 0.2 g/L and 0.4 g/L of white grape pomace extract, respectively; RGM: wine with red grape marc extract; WPP: wine with a wine-purified PS extract; CIY: wine with an inactivated commercial yeast. EE-SCFA: ethyl esters of straight-chain fatty acids; EE-BCFA: ethyl esters of branched-chain fatty acids.

**Table 2:** Volatile compounds analyzed in wine 2 and its treatments ^a^.

Compounds	Wine 2_C ^b^	Wine 2_WM	Wine 2_ WGP1	Wine 2_ WGP2	Wine 2_ RGM	Wine 2_WPP	Wine 2_CIY	Unit	*p*-Value
2-phenylethanol	19.0 ± 0.71 d	17.4 ± 0.45 bcd	16.1 ± 0.42 abc	16.3 ± 0.61 abc	17.7 ± 0.32 cd	15.8 ± 0.42 ab	15.3 ± 1.40 a	mg/L	**0.012**
1-propanol	39.0 ± 1.70 ab	35.6 ± 5.42 a	34.5 ± 4.01 a	36.9 ± 1.27 ab	39.9 ± 0.47 ab	74.9 ± 3.66 c	44.2 ± 1.82 b	mg/L	**0.000**
Isobutanol	70.5 ± 2.24	68.6 ± 4.36	68.5 ± 3.38	69.6 ± 0.24	69.3 ± 1.00	68.5 ± 1.26	69.6 ± 1.55	mg/L	0.967
1-butanol	2.02 ± 0.06 bc	2.42 ± 0.06 d	1.59 ± 0.17 a	2.02 ± 0.11 bc	1.86 ± 0.20 b	2.16 ± 0.03 cd	2.24 ± 0.01 cd	mg/L	**0.003**
2-methyl-1-butanol	31.6 ± 0.73	30.4 ± 1.33	30.8 ± 0.81	30.6 ± 0.39	30.4 ± 0.17	30.0 ± 1.18	30.3 ± 1.34	mg/L	0.707
3-methyl-1-butanol	129 ± 3.48	129 ± 4.18	133 ± 2.14	128 ± 2.33	129 ± 2.27	128 ± 0.36	129 ± 4.32	mg/L	0.661
Higher alcohols	291 ± 5.52 a	283 ± 14.9 a	285 ± 0.45 a	283 ± 1.62 a	288 ± 3.45 a	320 ± 6.84 b	291 ± 6.79 a	mg/L	**0.014**
Ethyl butyrate	174 ± 5.43 cd	176 ± 6.97 cd	184 ± 0.89 d	157 ± 4.18 a	160 ± 4.96 ab	168 ± 3.10 bc	159 ± 3.68 ab	µg/L	**0.004**
Ethyl hexanoate	193 ± 3.38 b	207 ± 2.35 c	212 ± 1.77 c	184 ± 8.54 ab	186 ± 5.21 ab	187 ± 1.15 ab	181 ± 4.85 a	µg/L	**0.001**
Ethyl octanoate	136 ± 1.90 abc	146 ± 0.47 c	146 ± 6.96 c	140 ± 11.6 bc	142 ± 0.59 bc	130 ± 1.83 ab	124 ± 1.59 a	µg/L	**0.029**
Ethyl decanoate	25.0 ± 0.09 b	28.2 ± 0.68 cd	28.2 ± 0.17 cd	27.4 ± 2.95 bcd	30.1 ± 0.07 d	26.7 ± 0.47 bc	20.7 ± 0.31 a	µg/L	**0.002**
Ethyl 2-methylbutyrate	3.09 ± 0.02 cd	4.10 ± 0.06 e	3.31 ± 0.02 d	2.75 ± 0.08 ab	2.61 ± 0.20 a	3.20 ± 0.12 cd	2.96 ± 0.16 bc	µg/L	**0.000**
Ethyl isovalerate	6.86 ± 0.06 ab	7.17 ± 0.33 bc	7.57 ± 0.09 c	6.49 ± 0.20 a	6.87 ± 0.43 ab	6.72 ± 0.17 ab	6.62 ± 0.09 a	µg/L	**0.028**
EE-SCFA ^c^	528 ± 10.8 bc	557 ± 9.12 cd	569 ± 4.13 d	509 ± 27.3 ab	517 ± 10.8 b	512 ± 0.34 ab	485 ± 9.82 a	µg/L	**0.004**
EE-BCFA ^c^ Branched esters	9.94 ± 0.04 a	11.3 ± 0.39 b	10.9 ± 0.11 b	9.24 ± 0.28 a	9.49 ± 0.63 a	9.93 ± 0.05 a	9.58 ± 0.07 a	µg/L	**0.002**
Isobutyl acetate	85.4 ± 6.76	84.2 ± 2.58	89.7 ± 0.57	75.3 ± 4.19	77.4 ± 2.39	86.2 ± 0.12	80.6 ± 5.42	µg/L	0.063
Butyl acetate	3.32 ± 0.31	3.37 ± 0.11	3.27 ± 0.17	2.86 ± 0.03	2.88 ± 0.18	3.05 ± 0.03	3.17 ± 0.18	µg/L	0.093
Isoamyl acetate	438 ± 4.11 b	464 ± 7.88 c	476.8 ± 2.64 c	396 ± 14.9 a	396 ± 14.1 a	415 ± 1.50 ab	404 ± 13.3 a	µg/L	**0.000**
Hexyl acetate	2.80 ± 0.19 cd	2.91 ± 0.04 d	2.89 ± 0.17 d	2.46 ± 0.06 ab	2.27 ± 0.13 a	2.60 ± 0.04 bc	2.57 ± 0.04 bc	µg/L	**0.005**
2-phenylethyl acetate	15.3 ± 0.12 a	18.2 ± 0.15 b	19.5 ± 1.40 b	15.27 ± 0.76 a	18.5 ± 2.06 b	14.0 ± 0.48 a	13.7 ± 0.58 a	µg/L	**0.004**
Alcohol acetates	545 ± 11.1 bc	573 ± 10.5 cd	592 ± 4.96 d	492 ± 19.9 a	496.6 ± 10.0 a	521 ± 0.84 ab	504 ± 19.2 a	µg/L	**0.001**
Linalool	7.09 ± 0.08	7.82 ± 0.02	8.16 ± 0.59	7.89 ± 1.12	7.65 ± 0.42	7.27 ± 0.14	6.79 ± 0.13	µg/L	0.222
α-terpineol	2.55 ± 0.05 a	3.05 ± 0.01 c	3.00 ± 0.34 bc	2.44 ± 0.11 a	2.60 ± 0.12 a	2.59 ± 0.12 a	2.66 ± 0.05 ab	µg/L	**0.032**
Citronellol	9.01 ± 0.11 ab	10.1 ± 0.20 c	9.88 ± 0.82 bc	9.04 ± 0.19 ab	9.11 ± 0.59 ab	8.62 ± 0.19 a	8.41 ± 0.28 a	µg/L	**0.036**
Geraniol	31.5 ± 0.13 cd	34.8 ± 0.26 e	32.4 ± 0.82 d	28.2 ± 0.77 a	27.5 ± 0.91 a	30.6 ± 0.39 bc	30.0 ± 0.36 b	µg/L	**0.000**
Terpenes	50.1 ± 0.11 ab	55.8 ± 0.49 c	53.5 ± 2.57 bc	47.6 ± 2.19 a	46.8 ± 2.05 a	49.1 ± 0.60 a	47.9 ± 0.00 a	µg/L	**0.005**
1-hexanol	1610 ± 13.0 c	1535 ± 28.3 bc	1429 ± 36.0 ab	1439 ± 59.9 ab	1408 ± 70.6 a	1554 ± 36.2 c	1517 ± 62.8 abc	µg/L	**0.028**
*trans*-3-hexen-1-ol	55.2 ± 0.80 d	50.0 ± 2.18 c	48.6 ± 1.85 bc	43.6 ± 0.03 a	44.7 ± 1.74 a	50.0 ± 0.26 c	46.7 ± 0.57 ab	µg/L	**0.001**
*cis*-3-hexen-1-ol	41.6 ± 1.09 d	37.5 ± 1.29 c	34.6 ± 2.55 b	30.0 ± 0.26 a	29.9 ± 0.30 a	37.4 ± 0.11 c	34.4 ± 0.55 b	µg/L	**0.000**
C6 Alcohols	1707 ± 14.9 c	1622 ± 31.7 bc	1512 ± 40.4 ab	1512 ± 60.1 ab	1483 ± 72.6 a	1641 ± 36.1 c	1598 ± 62.8 abc	µg/L	**0.020**
Isovaleric acid	269 ± 7.83 b	250 ± 4.37	252 ± 11.0	239 ± 16.4	244 ± 0.28	243 ± 1.18	236 ± 2.28	µg/L	0.058
Hexanoic acid	1057 ± 18.9 c	974 ± 20.7 b	943 ± 31.0 ab	923 ± 65.5 ab	951 ± 18.6 ab	931 ± 13.4 ab	883 ± 10.2 a	µg/L	**0.015**
Octanoic acid	772 ± 15.4 cd	781 ± 4.98 d	711 ± 3.34 abc	709 ± 52.0 abc	737 ± 43.4 bcd	679 ± 28.2 ab	657 ± 7.93 a	µg/L	**0.025**
Decanoic acid	187 ± 8.71 a	249 ± 23.3 cd	226 ± 21.1 bc	210 ± 0.21 ab	261 ± 2.13 d	185 ± 3.12 a	178 ± 21.4 a	µg/L	**0.004**
Dodecanoic acid	5.48 ± 0.16 b	8.67 ± 0.08 d	5.55 ± 0.12 b	4.52 ± 0.22 a	8.81 ± 0.30 d	6.19 ± 0.16 c	4.46 ± 0.09 a	µg/L	**0.000**
Fatty acids	2291 ± 33.2 d	2263 ± 6.78 d	2138 ± 60.0 bcd	2086 ± 134 abc	2202 ± 59.3 cd	2044 ± 46.1 ab	1958 ± 37.2 a	µg/L	**0.017**
Guaiacol	4.36 ± 0.09	4.18 ± 0.09	4.11 ± 0.50	3.89 ± 0.19	4.14 ± 0.30	4.25 ± 0.04	3.91 ± 0.02	µg/L	0.483
4-vinylguaiacol	61.2 ± 2.38	57.4 ± 1.23	64.8 ± 1.23	68.6 ± 1.58	78.6 ± 11.4	62.4 ± 5.54	61.6 ± 2.60	µg/L	0.054
Eugenol	16.5 ± 0.04 b	18.2 ± 0.45 c	15.6 ± 1.04 b	13.3 ± 0.34 a	13.5 ± 0.96 a	15.9 ± 0.17 b	15.3 ± 0.50 b	µg/L	**0.001**
Syringol	9.95 ± 0.02	9.16 ± 0.27	8.50 ± 0.82	8.47 ± 0.88	8.59 ± 0.08	9.25 ± 0.02	8.72 ± 0.28	µg/L	0.123
Volatile phenols	92.0 ± 2.23	88.9 ± 1.32	93.0 ± 3.58	94.2 ± 2.99	105 ± 12.6	91.7 ± 5.77	89.5 ± 3.40	µg/L	0.246
γ-butyrolactone	12,767 ± 403 d	11,903 ± 317 cd	10,943 ± 378 ab	10,821 ± 610 a	11,292 ± 214 abc	11,342 ± 279 abc	11,763 ± 232 bc	µg/L	**0.013**
γ-nonalactone	25.3 ± 0.18 ab	27.9 ± 0.41 bc	27.6 ± 1.89 bc	25.2 ± 0.53 ab	28.3 ± 2.13 c	24.4 ± 0.16 a	24.0 ± 0.73 a	µg/L	**0.030**
Lactones	12,792 ± 404 d	11,931 ± 317 cd	10,970 ± 380 ab	10,846 ± 610 a	11,320 ± 212 abc	11,367 ± 279 abc	11,787 ± 233 bc	µg/L	**0.013**
Vanillin	10.1 ± 0.28 a	10.2 ± 0.32 a	10.1 ± 0.44 a	10.2 ± 0.39 a	12.0 ± 0.08 b	10.8 ± 0.27 a	12.0 ± 0.49 b	µg/L	**0.002**
Methyl vanillate	4.75 ± 0.03	4.73 ± 0.10	4.76 ± 0.43	4.74 ± 0.48	4.86 ± 0.02	4.68 ± 0.10	4.40 ± 0.21	µg/L	0.733
Ethyl vanillate	367 ± 0.93	423 ± 13.0	389 ± 2.00	421 ± 58	406 ± 3.48	368 ± 5.32	340 ± 13.4	µg/L	0.062
Acetovanillone	28.5 ± 0.56	27.2 ± 0.94	26.7 ± 2.02	26.6 ± 1.78	27.9 ± 0.06	26.7 ± 0.01	25.6 ± 0.84	µg/L	0.338
Vanillin derivatives	411 ± 0.63	466 ± 12.3	431 ± 4.90	462 ± 61.0	450 ± 3.32	411 ± 4.96	382 ± 13.9	µg/L	0.074

Mean values ± standard deviation (*n* = 6). Values with different letters in each compound indicate statistically significant differences at *p* < 0.05 (marked in bold). C: wine control; WM: wine with white concentrated must extract; WGP1 and WGP2: wine with 0.2 g/L and 0.4 g/L of white grape pomace extract, respectively; RGM: wine with red grape marc extract; WPP: wine with a wine-purified PS extract; CIY: wine with an inactivated commercial yeast. EE-SCFA: ethyl esters of straight-chain fatty acids; EE-BCFA: ethyl esters of branched-chain fatty acids.

**Table 3:** Volatile compounds analyzed in wine 3 and its treatments ^a^.

Compounds	Wine 3_C ^b^	Wine 3_WM	Wine 3_ WGP1	Wine 3_ WGP2	Wine 3_ RGM	Wine 3_WPP	Wine 3_CIY	Unit	*p*-Value
2-phenylethanol	20.8 ± 0.02 c	19.1 ± 1.62 abc	20.3 ± 0.07 c	18.3 ± 0.85 ab	17.4 ± 0.30 a	20.7 ± 0.84 c	19.6 ± 0.21 bc	mg/L	**0.020**
1-propanol	26.9 ± 0.91	25.8 ± 0.36	25.7 ± 0.12	25.9 ± 0.19	26.0 ± 0.07	28.0 ± 1.55	25.7 ± 2.53	mg/L	0.462
Isobutanol	37.0 ± 0.33	36.5 ± 0.19	36.4 ± 0.45	35.1 ± 0.86	35.9 ± 0.29	37.9 ± 1.73	37.5 ± 3.83	mg/L	0.692
1-butanol	1.92 ± 0.04 e	1.84 ± 0.10 e	1.53 ± 0.03 c	1.37 ± 0.06 b	0.00 ± 0.00 a	2.25 ± 0.01 f	1.69 ± 0.06 d	mg/L	**0.000**
2-methyl-1-butanol	39.3 ± 0.35	38.6 ± 0.24	38.2 ± 0.14	37.0 ± 1.07	37.3 ± 0.11	40.8 ± 4.04	39.4 ± 6.00	mg/L	0.827
3-methyl-1-butanol	153 ± 0.87	153 ± 0.47	151 ± 1.81	149 ± 1.80	154 ± 3.05	163 ± 9.50	162 ± 8.86	mg/L	0.202
Higher alcohols	279 ± 2.43	275 ± 0.47	274 ± 2.42	267 ± 4.71	271 ± 2.99	292 ± 17.7	286 ± 21.5	mg/L	0.361
Ethyl butyrate	735 ± 33.8	815 ± 0.30	831 ± 40.7	737 ± 22.3	730 ± 16.9	754 ± 113	847 ± 47.2	µg/L	0.208
Ethyl hexanoate	636 ± 36.9 a	753 ± 17.9 c	747 ± 16.7 bc	687 ± 10.9 ab	687 ± 11.9 ab	708 ± 45.7 bc	754 ± 20.5 c	µg/L	**0.019**
Ethyl octanoate	579 ± 22.9 a	691 ± 40.2 b	684 ± 1.91 b	599 ± 2.08 a	625 ± 3.96 a	676 ± 28.7 b	714 ± 4.31 b	µg/L	**0.002**
Ethyl decanoate	126.5 ± 0.08 bcd	128 ± 10.7 cd	138 ± 1.24 d	104 ± 5.77 a	109 ± 9.89 ab	139 ± 11.4 d	119 ± 6.36 abc	µg/L	**0.017**
Ethyl 2-methylbutyrate	7.61 ± 0.94 ab	8.86 ± 0.20 c	9.26 ± 0.71 c	8.34 ± 0.25 abc	8.15 ± 0.09 abc	7.32 ± 0.23 a	8.53 ± 0.17 bc	µg/L	**0.044**
Ethyl isovalerate	20.6 ± 0.41 bc	22.2 ± 0.09 de	23.5 ± 1.29 e	21.0 ± 0.70 bcd	20.2 ± 0.38 b	18.6 ± 0.38 a	22.1 ± 0.63 cde	µg/L	**0.002**
EE-SCFA ^c^	2077 ± 93.4 a	2387 ± 68.6 c	2398 ± 56.8 c	2128 ± 25.3 ab	2152 ± 22.9 ab	2277 ± 119 bc	2433 ± 65.6 c	µg/L	**0.007**
EE-BCFA ^c^ Branched esters	28.2 ± 0.53 b	31.1 ± 0.29 cd	32.8 ± 2.00 d	29.3 ± 0.95 bc	28.3 ± 0.30 b	25.9 ± 0.61 a	30.6 ± 0.81 cd	µg/L	**0.003**
Isobutyl acetate	53.1 ± 2.64 a	55.6 ± 0.17 ab	60.5 ± 3.94 b	55.1 ± 2.24 bc	52.3 ± 0.44 bc	47.2 ± 1.33 bc	56.4 ± 2.66 c	µg/L	**0.014**
Butylacetate	5.49 ± 0.88 a	5.84 ± 0.22 ab	6.85 ± 0.46 bc	5.69 ± 0.33 a	6.13 ± 0.23 ab	6.17 ± 0.31 ab	7.52 ± 0.44 c	µg/L	**0.031**
Isoamyl acetate	1187 ± 42.4 a	1328 ± 12.0 bc	1355 ± 55.5 c	1227 ± 23.7 a	1225 ± 24.6 a	1239 ± 26.4 ab	1381 ± 72.9 c	µg/L	**0.013**
Hexyl acetate	14.2 ± 0.24	14.8 ± 0.45	16.7 ± 0.82	14.8 ± 0.20	14.5 ± 0.26	14.9 ± 1.28	15.5 ± 0.42	µg/L	0.065
2-phenylethyl acetate	30.4 ± 2.35	34.3 ± 4.50	38.5 ± 0.43	34.0 ± 2.58	32.5 ± 2.62	37.5 ± 0.90	42.5 ± 5.40	µg/L	0.068
Alcohol acetates	1290 ± 41.6 a	1438 ± 17.3 bc	1478 ± 60.3 c	1336 ± 19.0 ab	1331 ± 22.0 a	1345 ± 27.8 ab	1503 ± 81.8 c	µg/L	**0.011**
Linalool	3.77 ± 0.05 a	3.89 ± 0.12 a	4.14 ± 0.16 ab	3.64 ± 0.48 a	3.79 ± 0.16 a	4.15 ± 0.01 ab	4.72 ± 0.37 b	µg/L	**0.040**
α-terpineol	1.51 ± 0.06 a	1.86 ± 0.07 ab	2.11 ± 0.15 bc	1.74 ± 0.20 a	1.65 ± 0.08 a	2.23 ± 0.00 c	2.45 ± 0.29 c	µg/L	**0.004**
Citronellol	4.14 ± 0.11 a	5.12 ± 0.16 b	5.47 ± 0.26 bc	5.00 ± 0.38 b	4.85 ± 0.19 ab	5.51 ± 0.25 bc	6.03 ± 0.63 c	µg/L	**0.012**
Geraniol	2.29 ± 0.01 a	2.94 ± 0.33 bc	2.96 ± 0.12 bc	2.70 ± 0.17 ab	2.65 ± 0.07 ab	3.35 ± 0.15 cd	3.53 ± 0.37 d	µg/L	**0.009**
Terpenes	11.7 ± 0.13 a	13.8 ± 0.69 abc	14.7 ± 0.68 bcd	13.1 ± 1.24 ab	12.9 ± 0.36 ab	15.2 ± 0.42 cd	16.7 ± 1.66 d	µg/L	**0.011**
1-hexanol	1813 ± 69.7	1635 ± 152	1723 ± 107	1578 ± 30.1	1509 ± 52.1	1635 ± 65.9	1678 ± 155	µg/L	0.211
*trans*-3-hexen-1-ol	53.2 ± 2.59	47.1 ± 0.58	51.1 ± 2.16	46.4 ± 0.03	45.3 ± 0.28	45.3 ± 6.60	48.7 ± 3.18	µg/L	0.201
*cis*-3-hexen-1-ol	260 ± 6.36	228 ± 3.43	246 ± 7.79	224 ± 3.71	225 ± 3.00	225 ± 28.9	241 ± 14.6	µg/L	0.162
C6 Alcohols	2125 ± 78.6	1910 ± 156	2019 ± 117	1848 ± 33.8	1780 ± 55.4	1905 ± 30.5	1968 ± 173	µg/L	0.151
Isovaleric acid	986 ± 46.6 c	853 ± 4.95 a	941 ± 15.8 bc	857 ± 14.4 a	870 ± 20.9 ab	870 ± 58.1 ab	949 ± 47.8 bc	µg/L	**0.033**
Hexanoic acid	3059 ± 4.35 b	2703 ± 99.9 a	3030 ± 0.87 b	2513 ± 207 a	2591 ± 7.82 a	3102 ± 87.0 b	2957 ± 25.3 b	µg/L	**0.002**
Octanoic acid	2408 ± 150 a	2825 ± 62.5 bc	3146 ± 166 c	2498 ± 146 ab	2606 ± 13.2 ab	2775 ± 264 b	2803 ± 47.7 bc	µg/L	**0.019**
Decanoic acid	745 ± 27.7 a	911 ± 79.6 bc	893 ± 30.3 bc	802 ± 7.73 ab	852 ± 21.2 abc	960 ± 85.0 c	973 ± 92.4 c	µg/L	**0.048**
Dodecanoic acid	20.1 ± 0.44 a	26.7 ± 2.59 b	25.3 ± 0.84 b	20.4 ± 1.74 a	24.5 ± 1.46 b	30.2 ± 1.50 c	17.4 ± 0.18 a	µg/L	**0.001**
Fatty acids	7218 ± 71.4 b	7318 ± 250 bc	8035 ± 182 d	6689 ± 329 a	6944 ± 64.6 ab	7738 ± 152 cd	7698 ± 28.2 cd	µg/L	**0.002**
Guaiacol	4.26 ± 0.02	4.03 ± 0.19	4.27 ± 0.08	4.04 ± 0.10	3.84 ± 0.07	4.17 ± 0.25	4.25 ± 0.03	µg/L	0.084
4-vinylguaiacol	97.5 ± 9.30 d	64.4 ± 3.34 ab	81.0 ± 3.82 c	77.8 ± 6.01 bc	57.5 ± 6.94 a	75.4 ± 4.27 bc	75.9 ± 9.62 bc	µg/L	**0.010**
Eugenol	0.61 ± 0.06 a	0.98 ± 0.05 c	0.98 ± 0.00 c	0.80 ± 0.11 b	0.76 ± 0.06 ab	0.99 ± 0.10 c	1.41 ± 0.02 d	µg/L	**0.000**
Syringol	13.9 ± 0.02 ab	14.8 ± 1.00 bc	15.4 ± 0.06 cd	13.4 ± 0.14 a	12.8 ± 0.10 a	15.4 ± 0.39 cd	16.1 ± 0.56 d	µg/L	**0.001**
Volatile phenols	116 ± 9.36 d	84.2 ± 4.48 ab	102 ± 3.95 cd	96.0 ± 6.14 bc	75.0 ± 7.17 a	96.0 ± 4.81 bc	97.7 ± 9.00 bc	µg/L	**0.009**
γ-butyrolactone	15,165 ± 236 d	12,637 ± 463 bc	12,981 ± 320 c	11,928 ± 6.70 ab	11,542 ± 29.4 a	13,362 ± 483 c	13,030 ± 396 c	µg/L	**0.000**
γ-nonalactone	13.0 ± 0.75	14.1 ± 1.39	15.1 ± 0.09	13.8 ± 0.85	13.3 ± 0.73	15.6 ± 0.50	16.8 ± 1.82	µg/L	0.062
Lactones	15,178 ± 237 d	12,651 ± 464 bc	12,996 ± 320 c	11,942 ± 7.55 ab	11,555 ± 28.6 a	13,377 ± 484 c	13,047 ± 394 c	µg/L	**0.000**
Vanillin	14.5 ± 0.41 b	13.5 ± 0.05 ab	13.9 ± 0.27 b	16.9 ± 0.87 c	12.3 ± 0.29 a	16.2 ± 1.01 c	13.7 ± 0.20 b	µg/L	**0.001**
Methyl vanillate	1.84 ± 0.07 a	2.19 ± 0.09 bc	2.43 ± 0.00 d	2.07 ± 0.04 b	2.11 ± 0.08 b	2.34 ± 0.06 cd	2.47 ± 0.12 d	µg/L	**0.001**
Ethyl vanillate	156 ± 2.06 a	179 ± 15.2 abc	185 ± 2.89 bc	171 ± 10.8 ab	164 ± 7.19 ab	198 ± 13.1 c	194 ± 6.50 c	µg/L	**0.023**
Acetovanillone	37.0 ± 0.12 bc	35.9 ± 1.37 ab	38.4 ± 0.78 cd	34.8 ± 0.40 a	34.7 ± 0.05 a	38.6 ± 1.10 cd	39.7 ± 1.12 d	µg/L	**0.003**
Vanillin derivatives	210 ± 1.85 a	230 ± 16.7 abc	239 ± 2.38 bcd	225 ± 12.1 ab	213 ± 7.51 a	256 ± 11.0 d	250 ± 7.54 cd	µg/L	**0.014**

Mean values ± standard deviation (*n* = 6). Values with different letters in each compound indicate statistically significant differences at *p* < 0.05 (marked in bold). C: wine control; WM: wine with white concentrated must extract; WGP1 and WGP2: wine with 0.2 g/L and 0.4 g/L of white grape pomace extract, respectively; RGM: wine with red grape marc extract; WPP: wine with a wine-purified PS extract; CIY: wine with an inactivated commercial yeast. EE-SCFA: ethyl esters of straight-chain fatty acids; EE-BCFA: ethyl esters of branched-chain fatty acids.

## Data Availability

Data is contained within the article or Supplementary Material.

## Supplementary Materials

The following supporting information can be downloaded at: https://www.mdpi.com/article/10.3390/foods15091560/s1. Table S1. Range, linearity and correlation coefficient (R^2^) of the higher alcohols determined by GC-FID; Table S2. Range, linearity, correlation coefficient (R^2^), quantification ions and internal standards of the volatile compounds determined by GC-MS; Table S3. MANOVA of the volatile compounds of the wines studied; Table S4. ANOVA of the volatile compounds of the initial wines.

## Abbreviations

The following abbreviations are used in this manuscript:

PS	Polysaccharides
W1	Wine 1
W2	Wine 2
W3	Wine 3
PRAG	Polysaccharides rich in arabinose and galactose
AG	Arabinogalactans
Ar	Arabinans
AGP	Arabinogalactan-protein
RG-I	Rhamnogalacturonan I
RG-II	Rhamnogalacturonan II
HG	Homogalacturonan
NPP	Non-pectic polysaccharides
MP	Mannoproteins
GL	Glucans
CWM	Cell wall material
WM	Extract obtained from commercial white concentrated must
WGP	Extracts from white grape pomace
RGM	Extracts from red grape marc
WPP	Wine purified polysaccharide extract
CIY	Inactivated commercial yeast
GC-MS	Gas chromatography with mass spectrometry
FID	Flame ionization detector
HA	Higher alcohols
HA1	2-phenylethanol
HA2	1-propanol
HA3	Isobutanol
HA4	1-butanol
HA5	2-methyl-1-butanol
HA6	3-methyl-1-butanol
EE-SCFA	Ethyl esters of straight-chain fatty acids
EE1	Ethyl butyrate
EE2	Ethyl hexanoate
EE3	Ethyl octanoate
EE4	Ethyl decanoate
EE-BCFA	Ethyl esters of branched-chain fatty acids
EE5	Ethyl-2-methyl butyrate
EE6	Ethyl isovalerate
AA	Alcohol acetates
AA1	Isobutyl acetate
AA2	Butyl acetate
AA3	Isoamyl acetate
AA4	Hexyl acetate
AA5	2-phenylethyl acetate
A	Fatty acids
A1	Isovaleric acid
A2	Hexanoic acid
A3	Octanoic acid
A4	Decanoic acid
A5	Dodecanoic acid
TER	Terpenes
TER1	Linalool
TER2	α-terpineol
TER3	Citronellol
TER4	Geraniol
C6A	C6 Alcohols
C6A1	1-hexanol
C6A2	*Trans*-3-hexen-1-ol
C6A3	*Cis*-3-hexen-1-ol
VP	Volatile phenols
VP1	Guaiacol
VP2	4-vinylguaiacol
VP3	Eugenol
VP4	Syringol
LAC	Lactones
B.LAC	γ-butyrolactone
N.LAC	γ-nonalactone
VAN	Vanillin derivatives
VAN1	Vanillin
VAN2	Methyl vanillate
VAN3	Ethyl vanillate
VAN4	Acetovanillone
MANOVA	Multivariate analysis of variance
ANOVA	Analyses of variance
HSD	Tukey’s Honestly Significant Difference
PCA	Principal Component Analyses
GPA	General Procrustes Analyses
