# Supplementary material for: Volatile and Sensory Profiles of Young Red Wines Treated with Yeast and Grape Polysaccharides After Malolactic Fermentation
Source: Foods. 2026 May 1;15(9):1560. doi: 10.3390/foods15091560 (PMC13164142; doi:10.3390/foods15091560)
Supplement: Supplementary file 1 [file foods-15-01560-s001.zip › foods-4214875-supplementary.pdf]

foods

MDPI

Article

Volatile and sensory profiles of young red wines treated with yeast and grape polysaccharides after malolactic fermentation

María Curiel-Fernández[1], Estela Cano-Mozo[1], Belén Ayestarán[2], Zenaida Guadalupe[2], Thierry Doco[3], Silvia Pérez-Magariño[1,*]

Supplementary Material

Table S1. Range, linearity and correlation coefficient (R^2) of the higher alcohols determined by GC-FID.

Compounds	Range (mg/L)	Calibration [a]	R^2
Acetaldehyde	2.49 - 49.7	y = 0.117 * x + 0.249	0.998
Ethyl acetate	9.02 - 180	y = 0.154 * x + 0.183	0.996
Methanol	16.6 - 333	y = 0.098 * x - 0.014	0.999
1-Propanol	4.82 - 96.4	y = 0.230 * x - 0.220	0.999
Isobutanol	3.21 - 64.2	y = 0.316* x + 0.092	0.999
1-Butanol	0.810 - 16.2	y = 0.326 * x - 0.121	0.998
2-Methyl-1-butanol	4.08 - 81.5	y = 0.287 * x + 0.351	0.999
3-Methyl-1-butanol	20.2 - 405	y = 0.361* x + 0.162	0.999

[a] y: area; x: concentration

Table S2. Range, linearity, correlation coefficient (R^2), quantification ions and internal standards of the volatile compounds determined by GC-MS.

**Compounds**	**Range (μg/L)**	**Calibration** [a]	**R^2**	**Quantification ion (m/z)**	**Internal standard**
Isobutyl acetate	13.9-1392	y=0.00788*x-0.0123	0.991	TIC	Methyl octanoate
Ethyl butyrate	21.0-1400	y=0.00135*x-0.0036	0.993	88	Methyl octanoate
Ethyl 2-methyl-butyrate	1.73-173	y=0.00259*x-0.0018	0.994	57	Methyl octanoate
Ethyl isovalerate	3.46-345	y=0.00243*x-0.0055	0.994	88	Methyl octanoate
Butyl acetate	1.32-132	y=0.01070*x-0.0064	0.996	TIC	Methyl octanoate
Isoamyl acetate	49.0-4904	y=0.00844*x+0.3156	0.996	TIC	Methyl octanoate
Ethyl hexanoate	13.9-1.392	y=0.00290*x-0.0001	0.999	88	Methyl octanoate
Hexyl acetate	0.87-87.2	y=0.00229*x-0.0011	0.998	56	Methyl octanoate
1-hexanol	49.2-4920	y=0.00257*x-0.0910	0.992	56	3,4-dimethylphenol
trans-3-hexen-1-ol	6.52-261	y=0.00126*x+0.0004	0.998	41	3,4-dimethylphenol
cis-3-hexen-1-ol	13.6-542	y=0.00123*x-0.0009	0.998	67	3,4-dimethylphenol
Ethyl octanoate	13.9-1388	y=0.00414*x-0.0032	0.997	88	Methyl octanoate
Linalool	0.17-17.4	y=0.00302*x+0.0004	0.998	71	Methyl octanoate
γ–butyrolactone	224-56000	y=0.00019*x+0.0058	0.995	86	3,4-dimethylphenol
Ethyl decanoate	6.92-277	y=0.00203*x-0.0037	0.995	101	Methyl octanoate
Isovaleric acid	29.6-1184	y=0.00052*x-0.0057	0.998	60	3,4-dimethylphenol
α–terpineol	0.38-37.6	y=0.00189*x+0.0016	0.994	59	Methyl octanoate
Citronellol	0.17-17.2	y=0.00317*x+0.0007	0.998	41	Methyl octanoate
2-phenylethyl acetate	12.4-1240	y=0.00151*x+0.0243	0.996	91	Methyl octanoate
Hexanoic acid	74.2-3708	y=0.00167*x+0.1262	0.994	60	3,4-dimethylphenol
Geraniol	0.18-17.6	y=0.00548*x+0.0023	0.995	69	Methyl octanoate
Guaiacol	1.13-113	y=0.00764*x-0.0070	0.999	109	3,4-dimethylphenol
2-phenylethanol	326-32640	y=0.00806*x+8.5740	0.980	TIC	3,4-dimethylphenol
γ–nonalactone	0.20-39.0	y=0.00890*x+0.0030	0.997	85	Methyl octanoate
Octanoic acid	87.4-3494	y=0.01957*x+2.6831	0.995	TIC	3,4-dimethylphenol
Eugenol	1.07-107	y=0.00610*x+0.0065	0.997	164	Methyl octanoate
4-vinylguaiacol	8.00-800	y=0.00027*x+0.0010	0.996	135	Methyl octanoate
Syringol	3.96-396	y=0.00904*x+0.0184	0.999	154	3,4-dimethylphenol
Decanoic acid	16.0-1600	y=0.01330*x+0.5053	0.995	TIC	Methyl octanoate
Dodecanoic acid	2.00-160	y=0.00205*x+0.0033	0.997	73	Methyl octanoate
Vanillin	6.04-604	y=0.00598*x-0.0267	0.997	152	3,4-dimethylphenol
Methyl vanillate	0.80-80.0	y=0.01315*x+0.0138	0.991	151	3,4-dimethylphenol
Ethyl vanillate	4.00-400	y=0.00747*x+0.0267	0.997	151	Methyl octanoate
Acetovanillone	2.04-204	y=0.00516*x-0.0043	0.999	166	3,4-dimethylphenol

[a] y: area; x: concentration

Table S3. MANOVA of the volatile compounds of the wines studied.

	Wine type			Treatment			Wine type x Treatment			
**Compounds**	**F-ratio**	***p*-value** [a]	**% Wine type**[b]	**F-ratio**	***p*-value** [a]	**% Treatment**[b]	**F-ratio**	***p*-value** [a]	**% Interaction**[b]	**% residue**[b]
2-phenylethanol	43.6	**0.000**	46.90	2.10	0.096	6.79	5.42	**0.000**	35.01	11.3
1-propanol	181	**0.000**	46.43	21.5	**0.000**	16.58	22.3	**0.000**	34.30	2.70
Isobutanol	1,211	**0.000**	98.65	0.68	0.665	0.17	0.67	0.763	0.33	0.86
1-butanol	26.4	**0.000**	18.98	17.1	**0.000**	36.90	8.48	**0.000**	36.58	7.55
2-methyl-1-butanol	92.6	**0.000**	84.95	0.70	0.651	1.93	0.63	0.793	3.48	9.64
3-methyl-1-butanol	227	**0.000**	90.04	1.70	0.170	2.02	1.59	0.170	3.78	4.16
Higher alcohols	*11.4*	***0.001***	*24.36*	*5.27*	***0.002***	*34.86*	*1.33*	*0.274*	*17.61*	*23.2*
Ethyl butyrate	1,953	**0.000**	98.53	2.39	0.064	0.36	1.90	0.095	0.58	0.53
Ethyl hexanoate	5,366	**0.000**	98.73	8.27	**0.000**	0.46	5.62	**0.000**	0.62	0.19
Ethyl octanoate	7,910	**0.000**	98.51	12.7	**0.000**	0.47	11.9	**0.000**	0.89	0.13
Ethyl decanoate	2,075	**0.000**	96.86	7.51	**0.000**	1.05	5.69	**0.000**	1.59	0.49
Ethyl-2-methylbutyrate	1,673	**0.000**	96.21	9.46	**0.000**	1.63	4.51	**0.001**	1.56	0.60
Ethyl isovalerate	5,284	**0.000**	98.11	10.8	**0.000**	0.60	9.81	**0.000**	1.09	0.19
EE-SCFA [c]	*7,878*	**0.000**	*98.87*	*10.3*	**0.000**	*0.39*	*8.11*	**0.000**	*0.61*	*0.13*
EE-BCFA [c]	*5,148*	**0.000**	*97.93*	*13.6*	**0.000**	*0.78*	*9.61*	**0.000**	*1.10*	*0.20*
Isobutyl acetate	296	**0.000**	85.28	5.33	**0.002**	4.60	4.11	**0.002**	7.09	3.02
Butyl acetate	604	**0.000**	92.13	5.64	**0.001**	2.58	4.03	**0.003**	3.69	1.60
Isoamyl acetate	1,848	**0.000**	98.19	3.37	**0.017**	0.54	2.23	0.052	0.71	0.56
Hexyl acetate	5,196	**0.000**	99.17	5.31	**0.002**	0.30	2.87	**0.017**	0.33	0.20
2-phenylethyl acetate	487	**0.000**	89.90	4.76	**0.003**	2.64	4.99	**0.001**	5.52	1.94
Alcohol acetates	*1,781*	**0.000**	*97.89*	*4.07*	***0.007***	*0.67*	*2.60*	***0.026***	*0.86*	*0.58*
Linalool	429	**0.000**	92.13	2.62	**0.047**	1.69	3.04	**0.012**	3.92	2.26
α-terpineol	96.3	**0.000**	58.48	11.7	**0.000**	21.28	3.80	**0.004**	13.86	6.38
Citronellol	373	**0.000**	89.53	3.55	**0.014**	2.55	3.75	**0.004**	5.40	2.52
Geraniol	5,664	**0.000**	98.06	15.0	**0.000**	0.78	9.43	**0.000**	0.98	0.18
Terpenes	*2,525*	**0.000**	*97.42*	*7.60*	***0.002***	*0.88*	*5.59*	**0.000**	*1.29*	*0.41*
1-hexanol	31.1	**0.000**	47.08	2.48	0.057	11.25	2.83	**0.018**	25.76	15.9
trans-3-hexen-1-ol	0.28	0.758	0.53	4.59	**0.004**	26.12	4.70	**0.001**	53.46	19.9
cis-3-hexen-1-ol	3,318	**0.000**	99.04	3.20	**0.022**	0.29	2.01	0.078	0.36	0.31
C6 alcohols	*103*	**0.000**	*73.17*	*2.80*	***0.037***	*5.98*	*3.13*	***0.011***	*13.38*	*7.47*

https://doi.org/10.3390/xxxxx

Isovaleric acid	4,406	**0.000**	98.81	5.13	**0.002**	0.35	4.50	**0.001**	0.61	0.24
Hexanoic acid	4,814	**0.000**	97.47	14.9	**0.000**	0.90	11.6	**0.000**	1.41	0.21
Octanoic acid	2,311	**0.000**	97.45	5.61	**0.001**	0.71	5.53	**0.000**	1.40	0.44
Decanoic acid	1,584	**0.000**	96.97	4.21	**0.006**	0.77	4.40	**0.002**	1.62	0.64
Dodecanoic acid	1,450	**0.000**	87.64	41.4	**0.000**	7.50	11.7	**0.000**	4.23	0.63
Fatty acids	*8,684*	***0.000***	*98.65*	*11.7*	***0.000***	*0.40*	*12.2*	***0.000***	*0.83*	*0.12*
Guaiacol	4.61	**0.022**	13.31	2.39	0.065	20.68	2.06	0.071	35.66	30.4
4-vinylguaiacol	29.2	**0.000**	30.34	2.99	0.287	9.32	7.91	**0.000**	49.41	10.9
Eugenol	3,248	**0.000**	97.10	12.9	**0.000**	1.15	7.98	**0.000**	1.43	0.31
Syringol	873	**0.000**	90.63	13.2	**0.000**	4.10	6.70	**0.000**	4.17	1.09
Volatile phenols	*11.1*	***0.001***	*15.42*	*2.99*	***0.029***	*12.41*	*6.94*	***0.000***	*57.65*	*14.5*
γ-butyrolactone	79.3	**0.000**	45.96	11.8	**0.000**	20.55	7.88	**0.000**	27.41	6.09
γ-nonalactone	424	**0.000**	90.82	3.00	**0.028**	1.93	3.90	**0.003**	5.01	2.25
Lactones	*78.2*	***0.000***	*45.64*	*11.8*	***0.000***	*20.63*	*7.88*	***0.000***	*27.60*	*6.13*
Vanillin	139	**0.000**	62.35	5.04	**0.002**	6.80	9.68	**0.000**	26.12	4.72
Methyl vanillate	654	**0.000**	93.79	4.50	**0.004**	1.94	3.21	**0.009**	2.77	1.51
Ethyl vanillate	725	**0.000**	94.41	4.06	**0.007**	1.59	3.37	**0.007**	2.63	1.37
Acetovanillone	603	**0.000**	89.59	6.27	**0.001**	2.79	6.79	**0.000**	6.05	1.56
Vanillin derivatives	*634*	***0.000***	*93.50*	*3.91*	***0.009***	*1.73*	*3.64*	***0.005***	*3.22*	*1.55*

[a] Values in bold show statistically significant differences in each compound and factor considered (*p*-values <0.05). [b] Percentage of attributable variance of the independent effect of wine type and treatment, the interaction wine type and treatment and residue. [c] EE-SCFA: ethyl esters of straight-chain fatty acids; EE-BCFA: ethyl esters of branched-chain fatty acids.

Table S4. ANOVA of the volatile compounds of the initial wines.

Compounds	W1_C	W2_C	W3_C	Unit	p-value [a]
2-phenylethanol	16.4±0.48 a	16.3±0.57 a	19.3±0.32 b	mg/L	**0.013**
1-propanol	31.9±0.00 b	34.5±0.15 c	22.3±0.88 a	mg/L	**0.000**
Isobutanol	66.4±0.44 b	67.1±2.55 b	39.4±0.42 a	mg/L	**0.001**
2-methyl-1-butanol	27.1±0.09 a	29.2±0.62 b	39.0±0.88 c	mg/L	**0.001**
3-methyl-1-butanol	129±1.43 a	127±3.88 a	171±3.06 b	mg/L	**0.001**
Higher alcohols	*271±2.26*	*274±7.77*	*291±5.56*	mg/L	0.074
Ethyl butyrate	156±9.07 a	151±2.75 a	733±12.7 b	μg/L	**0.000**
Ethyl hexanoate	177±9.40 a	177±1.09 a	673±5.28 b	μg/L	**0.000**
Ethyl octanoate	146±4.54 a	137±3.56 a	692±28.7 b	μg/L	**0.000**
Ethyl decanoate	30.4±1.36 a	29.4±1.94 a	149±2.92 b	μg/L	**0.000**
Ethyl 2-methylbutyr-	2.03±0.03 a	2.16±0.01 a	6.47±0.47 b	μg/L	**0.001**
Ethyl isovalerate	5.75±0.04 a	6.22±0.00 a	19.1±1.21 b	μg/L	**0.001**
EE-SCFA [b]	*510±21.7 a*	*495±3.84 a*	*2247±18.3 b*	μg/L	**0.000**
EE-BCFA [b]	*7.78±0.01 a*	*8.38±0.01 a*	*25.5±1.68 b*	μg/L	**0.001**
Isobutyl acetate	72.1±4.36 b	67.9±3.82 b	47.5±0.58 a	μg/L	**0.010**
Butylacetate	2.41±0.10 a	2.62±0.04 a	5.28±0.24 b	μg/L	**0.001**
Isoamyl acetate	320±20.4 a	348±0.40 a	1237±36.8 b	μg/L	**0.000**
Hexyl acetate	2.12±0.16 a	2.37±0.05 a	17.2±0.29 b	μg/L	**0.000**
2-phenylethyl acetate	17.1±1.07 a	19.8±0.06 a	46.2±3.30 b	μg/L	**0.001**
Alcohol acetates	*414±23.7 a*	*441±4.18 a*	*1353±40.7 b*	μg/L	**0.000**
Linalool	6.37±0.01 b	6.52±0.08 b	4.15±0.10 a	μg/L	**0.000**
α-terpineol	1.75±0.17	1.90±0.14	1.55±0.13	μg/L	0.203
Citronellol	7.14±0.50 b	7.69±0.07 b	4.61±0.25 a	μg/L	**0.005**
Geraniol	27.2±1.85 b	32.8±0.95 b	3.81±0.13 a	μg/L	**0.000**
Terpenes	*42.4±2.54 b*	*48.9±1.25 c*	*14.1±0.15 a*	μg/L	**0.000**
1-hexanol	1079±14.1	1375±116	1187±88.9	μg/L	0.085
trans-3-hexen-1-ol	34.0±2.26	35.3±1.47	36.6±2.22	μg/L	0.510
cis-3-hexen-1-ol	27.0±1.71 a	32.0±1.87 a	229±12.8 b	μg/L	**0.000**
C6 Alcohols	*1140±10.2*	*1443±120*	*1453±73.8*	μg/L	0.050
Isovaleric acid	210±10.7 a	238±10.8 a	866±31.7 b	μg/L	**0.000**
Hexanoic acid	804±24.3 a	810±21.3 a	2706±55.0 b	μg/L	**0.000**
Octanoic acid	561±21.9 a	514±2.65 a	3114±243 a	μg/L	**0.001**
Decanoic acid	240±9.65 a	222±4.80 a	1023±55.6 b	μg/L	**0.000**
Dodecanoic acid	7.56±0.45 a	8.53±0.05 a	31.83±2.21 b	μg/L	**0.001**
Fatty acids	*1822±47.6 a*	*1792±39.6 a*	*7742±388 b*	μg/L	**0.000**
Guaiacol	3.35±0.04	3.64±0.25	4.34±0.37	μg/L	0.068
4-vinylguaiacol	83.1±1.94 a	102±8.41 b	116±1.83 b	μg/L	**0.017**
Eugenol	12.8±0.77 b	18.0±0.39 c	1.31±0.03 a	μg/L	**0.000**
Syringol	6.83±0.37 a	7.04±0.30 a	16.2±0.53 b	μg/L	**0.000**
Volatile phenols	*106±2.30 a*	*131±9.35 b*	*138±0.96 b*	μg/L	**0.022**
γ-butyrolactone	8098±183 a	8286±5.18 a	10868±513 b	μg/L	0.005
γ-nonalactone	27.1±1.95 b	29.5±0.01 b	16.8±0.03 a	μg/L	**0.003**
Lactones	*8125±181 a*	*8316±5.16 a*	*10885±513 b*	μg/L	0.005
Vanillin	13.4±0.18 a	11.4±0.79 a	21.4±1.09 b	μg/L	**0.002**

 https://doi.org/10.3390/xxxxx

Methyl vanillate	4.00±0.15 b	4.42±0.29 b	2.89±0.11 a	μg/L	**0.010**
Ethyl vanillate	403±13.8 c	335±10.7 b	227±18.3 a	μg/L	**0.003**
Acetovanillone	25.2±0.23 a	26.8±0.58 a	44.0±1.84 b	μg/L	**0.001**
Vanillin derivatives	*446±14.3 c*	*378±9.01 b*	*295±15.2 a*	μg/L	**0.003**

[a] Values in bold show statistically significant differences in each compound and factor considered (p-values <0.05). [b] EE-SCFA: ethyl esters of straight-chain fatty acids; EE-BCFA: ethyl esters of branched-chain fatty acids.
